# Tracing mitochondrial marks of neuronal aging in iPSCs-derived neurons and directly converted neurons

**DOI:** 10.1038/s42003-025-08152-2

**Published:** 2025-05-10

**Authors:** Nimmy Varghese, Leonora Szabo, M. Zameel Cader, Imane Lejri, Amandine Grimm, Anne Eckert

**Affiliations:** 1 Research Cluster Molecular and Cognitive Neurosciences, University of Basel, Basel, Switzerland; 2 Neurobiology Lab for Brain Aging and Mental Health, University Psychiatric Clinics Basel, Basel, Switzerland; 3 Nuffield Department of Clinical Neuroscience, University of Oxford, Oxford, UK; 4 Department of Biomedicine, University of Basel, Basel, Switzerland; 5 Present Address: Max Planck Florida Institute for Neuroscience, Jupiter, FL USA

**Keywords:** Neural ageing, Energy metabolism

## Abstract

This study aims to determine if neurons derived from induced pluripotent stem cells (iPSCsNs) and directly converted neurons (iNs) from the same source cells exhibit changes in mitochondrial properties related to aging. This research addresses the uncertainty around whether aged iPSCsNs retain aging-associated mitochondrial impairments upon transitioning through pluripotency while direct conversion maintains these impairments. We observe that both aged models exhibit characteristics of aging, such as decreased ATP, mitochondrial membrane potential, respiration, NAD^+^/NADH ratio, and increased radicals and mitochondrial mass. In addition, both neuronal models show a fragmented mitochondrial network. However, aged iPSCsNs do not exhibit a metabolic shift towards glycolysis, unlike aged iNs. Furthermore, mRNA expression differed significantly between aged iPSCsNs and aged iNs. The study concludes that aged iPSCsNs may differ in transcriptomics and the aging-associated glycolytic shift but can be a valuable tool for studying specific feature of mitochondrial neuronal aging in vitro alongside aged iNs.

## Abstract (web summary)

Side-by-side modeling of iPSC-derived neurons (iPSCsNs) and directly converted neurons (iNs) from the same aged donor shows both retain mitochondrial aging traits, though iPSCsNs exhibit partial rejuvenation in transcriptomics and metabolic shift.

## Introduction

Understanding molecular and cellular aging is still inadequate, particularly in the human brain. Nonetheless, a comprehensive understanding of the biological processes behind aging is crucial for optimal brain health^1^. One of the most significant impairments that occur in aging is energy imbalance and deficit^2^. Mitochondria are essential for sustaining life by facilitating energy conversion processes within cells. Consequently, impaired mitochondrial functioning can severely disrupt cellular energy balance, with neurons particularly vulnerable to aging due to their reliance on mitochondria^3–6^. Gaining insight into the mechanisms behind mitochondrial malfunction of age-related metabolic disorders has significant potential for advancing our understanding of aging-associated adverse health issues. One major challenge is the identification and utilization of suitable in vitro models that accurately mimic human brain aging. However, practical in vitro models for neuronal aging are limited^7,8^. In recent years, novel approaches have emerged, generating neurons through nuclear reprogramming of somatic cells^9,10^. These approaches include the advanced neuronal models of induced pluripotent stem cell-derived neurons (iPSCsNs)^11^ and directly converted neurons (iNs) from human fibroblasts (HFs)^12,13^. These reprogramming technologies opened the possibility of obtaining neurons from more easily accessible sources. Nevertheless, research budgets are often limited, making time and budget crucial when choosing cellular models^14,15^. In contrast, iPSCsNs require a prolonged generation time by transiting an embryonic-like state. Once established, iPSCs can expand indefinitely, providing an abundant cell supply^10^, preferable for conducting bioenergetic investigations, drug screening, and transplantation requiring considerable material. Conversely, generating iNs takes fewer weeks but is limited by the number of HFs used, making extensive screenings challenging. Nevertheless, a key consideration point is the preservation of the aging-associated phenotype. In this regard, iPSCs technology appears to be constrained by cellular rejuvenation, leading to a reset of age indicators associated with the source cells^9,14,16–20^. By bypassing the self-renewal and pluripotent stages, iNs are considered to preserve their age-associated hallmarks from their initial cell source^12,13,16,19,21–23^. Nonetheless, recent studies have speculated the extent to which iPSCs and their derived cells really lose the aging donor signature^24,25^. Still, evidence of mitochondrial impairments, especially at the functional level, has not been reported yet. Our previous study^26^ demonstrated that aging-associated phenotypes on the mitochondrial level were retained in aged iPSCs. Therefore, aged iPSCsNs could also represent an aging phenotype alongside aged iNs. The primary objective of this study was to examine the extent to which the advanced neuronal models of iPSCsNs and iNs derived from the same individuals accurately represent aging-related characteristics at the mitochondrial level.

## Results

### Assessing the mitochondrial impairment in HFs

**Fig. 1:**
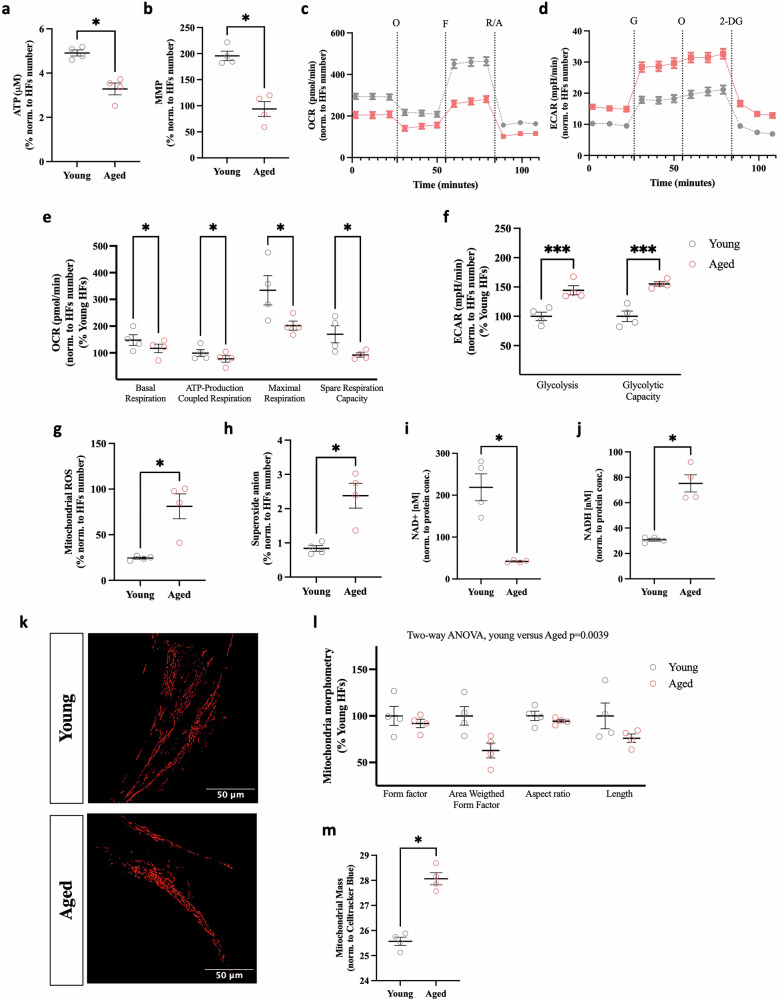
Aging phenotype in aged HFs in comparison of young HFs on mitochondrial properties. **a** Cellular ATP level comparing HFs from aged and young donors using a bioluminescence assay (*N* = 5 independent experiments, *n* = 13–14 replicates per experiment for each donor). **b** MMP level comparing aged HFs to young HFs by staining with TMRM. The fluorescence was detected at ex: 548 nm/em: 574 nm (*N* = 5 independent experiments, *n* = 10–17 replicates per experiment for each donor). **c** Mito Stress Test profile representing the OCR of young and aged HFs after sequential injection of oligomycin (O, 1 µM), FCCP (F, 2 µM), and lastly combined rotenone (R, 2 µM) with antimycin A (A, 4 µM). **d** Glycolysis Stress Test profile exhibiting the ECAR of aged and young HFs after sequential injection of glucose (G, 10 mM), Oligomycin (O, 1 µM), and lastly 2-deoxy-glucose (2-DG, 25 mM). **e** Bioenergetic parameters calculated from the Agilent Seahorse XF Cell Mito Stress Test of young and aged HFs. Basal respiration, ATP-production coupled respiration, maximal respiration, and spare respiration capacity (*N* = 5–6 independent experiments, *n* = 3–5 replicates per experiment for each donor). **f** Bioenergetic parameters were determined from the Agilent Seahorse XF Glycolysis Stress Test by comparing young and aged HFs. Glycolysis and glycolytic capacity (*N* = 4–6 independent experiments, *n* = 2–4 replicates per experiment for each donor). **g** Mitochondrial superoxide anion using the MitoSOX dye to compare young and aged HFs. The fluorescence was detected at ex: 485 nm/em: 535 nm (*N* = 5 independent experiments, *n* = 11–12 replicates per experiment for each donor). **h** Mitochondrial ROS detection in aged and young HFs. (ex: 485 nm/em: 535 nm). The fluorescence was detected at ex: 531 nm/em: 595 nm (*N* = 5 independent experiments, *n* = 11–12 replicates per experiment for each donor). Cellular NAD+ content (**i**) and NADH content (**j**) from young and aged HFs represented as normalized values to the protein concentration (*N* = 5 independent experiments, *n* = 3 replicates per experiment for each donor). **k**, **l** Mitochondrial network morphology was assessed in HFs from young and aged human donors by staining the mitochondria with TOMM20 (**k**). Calculated mitochondrial parameters (**l**) Form Factor Area Weighted Form Factor, Aspect Ratio, and Length (*N* = 4–5 independent experiments, *n* = 12–33 replicates per experiment for each donor). **m** Mitochondrial Mass comparing young and aged HFs assessed by using the MitoTracker™ Green FM (ex: 490 nm/em: 516 nm) to stain mitochondria and were normalized to the cell area using CellTracker™ Blue CMAC Dye (ex: 353 nm/em: 466 nm) (*N* = 4 independent experiments, *n* = 15–16 replicates per experiment for each donor). Data information: All data are represented as the mean ± SEM of each four different young and aged HFs. Statistical parameters, including the number of values, minimum, maximum, range, mean, standard deviation, and standard error of the mean, are presented in Supplementary Data 2. Values were normalized on the cell count. Non-parametric Mann–Whitney test was performed to compare young HFs versus aged HFs (*p < 0.05, **p < 0.01, ***p < 0.001) or two-way ANOVA was applied to compare multiple parameters.

We first analyzed the aging signatures of the corresponding donor HFs (Young: Age_mean_ = 31 years, SD = 5.03 and Aged: Age_mean_ = 69 years, SD = 7.632). Foremost, we measured the cellular adenosine triphosphate (ATP) level (Fig. 1a) to determine the cellular metabolic activity. We found a significant decline in the overall ATP level in the aged HFs compared to the young. Next, we detected the mitochondrial membrane potential (MMP), the ATPase driving force in oxidative phosphorylation (OXPHOS)^27^. The MMP (Fig. 1b) in aged HFs dropped compared to the young HFs. To receive a precise image of the mitochondrial respiratory activity, the oxygen consumption rate (OCR) was measured by performing the Seahorse XF Cell Mito Stress Test (Fig. 1c, e). The aged HFs represented a lower respiratory rate (Fig. 1c). All determined mitochondrial bioenergetic parameters (Fig. 1e) calculated from the OCR profile, basal respiration, ATP-production coupled respiration, maximal respiration, and spare respiration capacity indicated a significant decrease in aged HFs compared to young HFs. Besides mitochondrial respiration, glycolysis, the second cellular energy source^28^, was assessed using the Seahorse XF Glycolysis Stress Test Kit (Fig. 1d, f). By sequential injection of distinctive molecules, the glycolysis and glycolysis capacity were determined by measuring the extracellular acidification rate (ECAR). We observed a substantial increase in all glycolytic parameters in aged HFs. Next, we quantified the mitochondrial superoxide anion radicals (Fig. 1g) and mitochondrial ROS (Fig. 1h), where we observed a substantial increase in both mitochondrial free radicals in aged HFs. Our investigation on NAD revealed that the concentration of NAD^+^ decreased (Fig. 1i) while the concentration of NADH increased (Fig. 1j) in aged HFs. The NAD^+^/NADH ratio decreased from 7.1 in young HFs to 0.56 in aged HFs (Supplementary Data 1). The mitochondrial network morphology was quantified further by visualizing the mitochondria using a TOMM20 staining. This analysis showed significant differences in all calculated parameters between aged and young (Fig. 1l), where aged HFs exhibited a more fragmented mitochondrial state. This was indicated in Fig. 1l by a lower Form Factor (FF), Area Weighted Form Factor (AW), Aspect Ratio (AR), and shorter mitochondrial length, signifying a more circular mitochondrial morphology in age. As assessed by two-way ANOVA, the mitochondrial morphometry analysis revealed a significant difference between young and aged groups, with a *p* value of 0.0039. Upon visual comparison (Fig. 1k), young HFs appeared to have a more tubular or elongated morphology. In contrast, aged HFs exhibited a more fragmented mitochondrial network. Subsequently, the mitochondrial mass (Fig. 1m) was evaluated to observe the buildup of malfunctioning mitochondria. We observed an upturn in mitochondrial mass in age.

### Investigation of mitochondrial impairments in aged iNs

**Fig. 2:**
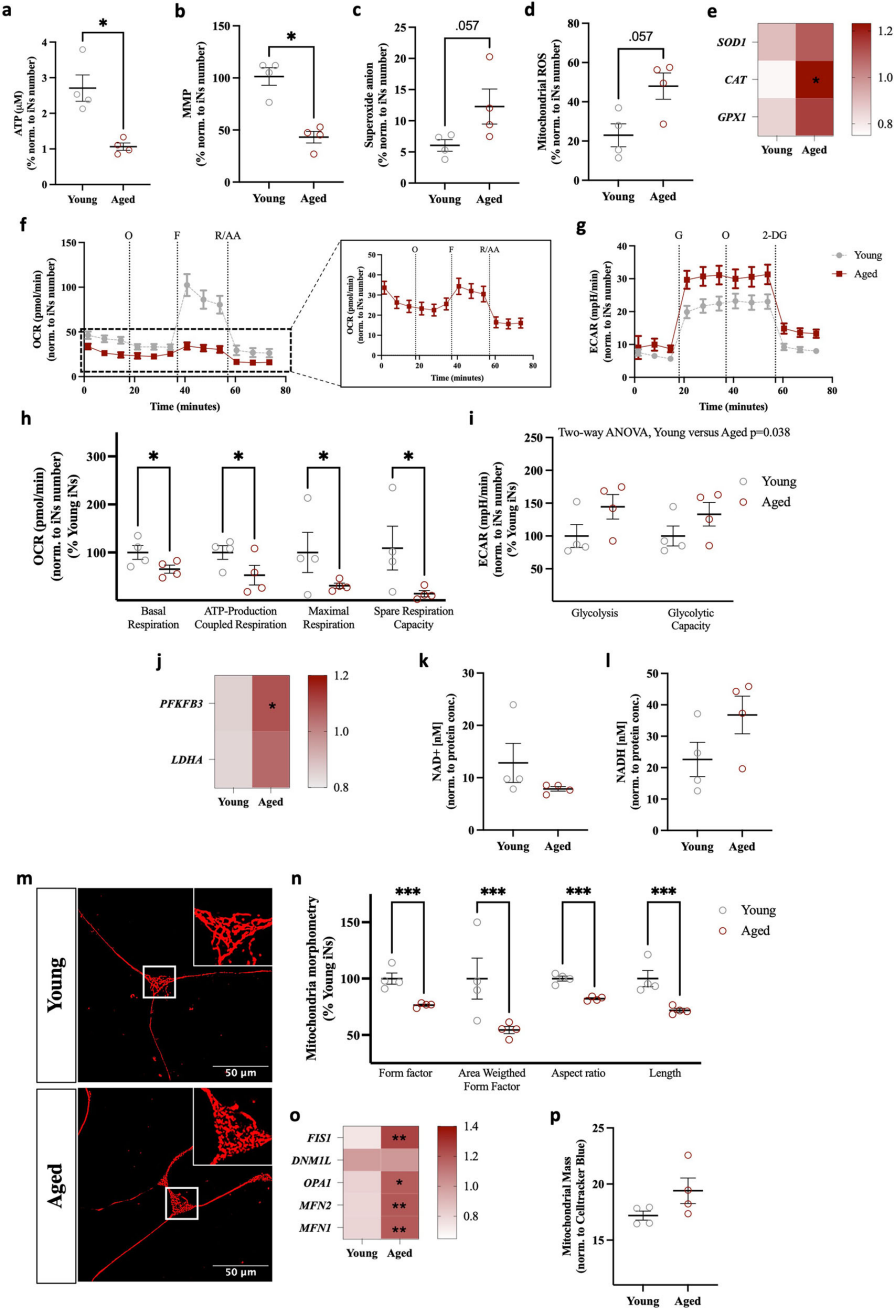
Aged iNs represented aging-associated phenotype from the donor on the mitochondrial level and the correlated metabolic shift toward anaerobic glycolysis. **a** Cellular ATP level comparing iNs from aged and young human donors (*N* = 3–4 independent experiments, *n* = 2–3 replicates per experiment for each donor). **b** MMP level was measured in aged iNs and young iNs by staining them with TMRM. The fluorescence was detected at ex: 548 nm/em: 574 nm (*N* = 4–5 independent experiments, *n* = 2–3 replicates per experiment). **c** Mitochondrial superoxide anion detection using the MitoSOX dye to compare young and aged iNs. The fluorescence was detected at ex: 485 nm/em: 535 nm (*N* = 4–5 independent experiments, *n* = 2–3 replicates per experiment for each donor). **d** Mitochondrial ROS detection in aged and young HFs. (ex: 485 nm/em: 535 nm). The fluorescence was detected at ex: 531 nm/em: 595 nm (*N* = 4–5 independent experiments, *n* = 2–3 replicates per experiment for each donor). **e** Relative gene expression of relevant anti-oxidative stress enzymes, *SOD1, CAT*, and *GPX1*. The data are represented as gene expression (2 ^(−Avg.(Delta(Ct))^) as the Housekeeping gene *GAPDH* was utilized. **f** Mito Stress Test profile representing the OCR of young and aged iNs after sequential injection of oligomycin (O, 2,5 µM), FCCP (F, 2 µM), and lastly combined rotenone (R, 2 µM) with antimycin A (A, 2 µM). **g** Glycolysis Stress Test profile representing the ECAR of aged and young iNs after sequential injection of glucose (G, 10 mM), Oligomycin (O, 1 µM), and lastly, 2-deoxy-glucose (2-DG, 25 mM). **h** Bioenergetic parameters of the mitochondria of young and aged iNs. Basal respiration, ATP-production coupled respiration, proton leak, maximal respiration, and spare respiration capacity (*N* = 4–5 independent experiments, *n* = 2–3 replicates per experiment). **i** Bioenergetic parameters of glycolysis comparing young and aged iNs. Glycolysis, glycolytic capacity, and glycolytic reverse (*N* = 3–5 independent experiments, *n* = 2–3 replicates per experiment for each donor). **j** Relative gene expression of relevant glycolysis-regulating genes, *PFKFB3* and *LDHA*. The data are represented as gene expression (2 ^(−Avg.(Delta(Ct))^) as the Housekeeping gene *GAPDH* was utilized. **k**, **l** Cellular NAD^+^ content (L) and NADH (M) content from young and aged iNs represented as normalized values to the protein concentration (*N* = 4–5 independent experiments, *n* = 2–3 replicates per experiment for each donor). **m**, **n** The mitochondrial network morphology (**m**) visualization was assessed in iNs from young and aged human donors by staining the mitochondria with TOMM20. Calculated mitochondrial parameters (**n**) Form Factor, Area Weighted Form Factor, Aspect Ratio, and Length (*N* = 4–5 independent experiments, *n* = 12–33 replicates per experiment for each donor). **o** Relative gene expression of relevant genes involved in mitochondrial dynamics: *FIS1, DNM1L, OPA1*, *MFN2*, and *MFN1*. The data are represented as gene expression (2 ^(−Avg.(Delta(Ct))^) as the Housekeeping gene *GAPDH* was utilized. **p** Mitochondrial Mass comparing young and aged iNs assessed by using the MitoTracker™ Green FM (ex: 490 nm/em: 516 nm) to stain mitochondria and were normalized to the cell area using Celltracker blue (ex: 353 nm/em: 466 nm) (*N* = 4–5 independent experiments, *n* = 2–3 replicates per experiment for each donor). Data information: All data are represented as the mean ± SEM of each four different young and aged iNs. Statistical parameters, including the number of values, minimum, maximum, range, mean, standard deviation, and standard error of the mean, are presented in Supplementary Data 2. Only three donors were assessed for gene expression using three technical replicates. Values were normalized on the cell count. The representative images were chosen for visualization purposes. Non-parametric Mann–Whitney test was performed to compare young iNs versus aged iNs (*p < 0.05, **p < 0.01, ***p < 0.001), or a two-way ANOVA was applied to compare multiple parameters.

Next, we assessed whether aged iNs served as a reliable in vitro model system for neuronal aging. First, an investigation was conducted on the bioenergetic parameters, revealing a decrease in cellular ATP levels (Fig. 2a) in aged iNs when compared to young iNs. Upon MMP examination (Fig. 2b), a significant decline was observed in aged iNs. Furthermore, mitochondrial superoxide anion radicals (Fig. 2c) and mitochondrial ROS (Fig. 2d) showed a clear trend toward an increase in aged iNs compared to young iNs, with a *p* value of 0.057. When analyzing individual data points, both mitochondrial superoxide anion radicals and mitochondrial ROS showed a significant age-related increase (Fig. S2c, d). Following this, the mRNA expression of antioxidant defense mechanism-related proteins, namely superoxide dismutase 1 (SOD1), catalase (CAT), and glutathione peroxidase (GPX1), were examined (Fig. 2e). The findings revealed no statistically significant disparities in the mRNA expression of *SOD1* and *GPX1* between aged and young iNs. Nevertheless, the *CAT* expression was markedly increased in aged iNs. Afterward, the Seahorse XF Cell Mito Stress Test Kit (Fig. 2f) was conducted, representing a significant decrease in the calculated mitochondrial bioenergetic parameters in aged iNs (Fig. 2h). Examining the Glycolysis (Fig. 2g, i), we observed that aged iNs demonstrated a notable elevation in glycolysis and glycolytic capacity compared to young iNs. To gain valuable insights into this intricate system, we analyzed the glycolysis-related gene expression of two critical enzymes, 6-phosphofructo-2-kinase/fructose-2, 6-bisphosphatase-3 (PFKFB3) and lactate dehydrogenase A (*LDHA*). While studying the *PFKF3B* and *LDHA* gene expression (Fig. 2j), we observed that the *PFKF3B* expression was significantly upregulated in aged iNs. In contrast, the *LDHA* expression showed no significant differences. Analysis of the NAD⁺/NADH redox state revealed that aged iNs exhibited a decline in NAD⁺ levels (Fig. 2k) alongside an increase in NADH levels (Fig. 2l), although these changes were not statistically significant. Nevertheless, this shift was accompanied by a notable reduction in the NAD⁺/NADH ratio, decreasing from 0.53 in young iNs to 0.22 in aged iNs (Supplementary Data 1). While examining the mitochondrial network (Fig. 2m, n), we observed that aged iNs exhibited a more fragmented mitochondrial morphology than young iNs. In the mitochondrial morphometry (Fig. 2n), a decrease in FF was observed alongside a reduction in AW and AR, suggesting a more round and circular mitochondrial morphology in aged iNs. In addition, the mitochondria in aged iNs exhibit reduced length. After a visual analysis (Fig. 2m), a noticeable fragmentation was observed within aged iNs. By analyzing the gene expression of mitochondrial dynamics proteins, we detected an increase in the gene expression responsible for mitochondrial fusion and fission (Fig. 2o). More precisely, we observed a significant upregulation of *FIS1*, essential for fission, and *MFN1*, *MFN2*, and *OPA1*, crucial for fusion, in aged iNs compared to young. Additionally, a tendency toward elevated mitochondrial mass was observed in aged iNs (Fig. 2p). Notably, when comparing individual data points, this increase reached statistical significance (Fig. S2i).

### Examination of mitochondrial impairment of iPSCsNs of aged individuals

**Fig. 3:**
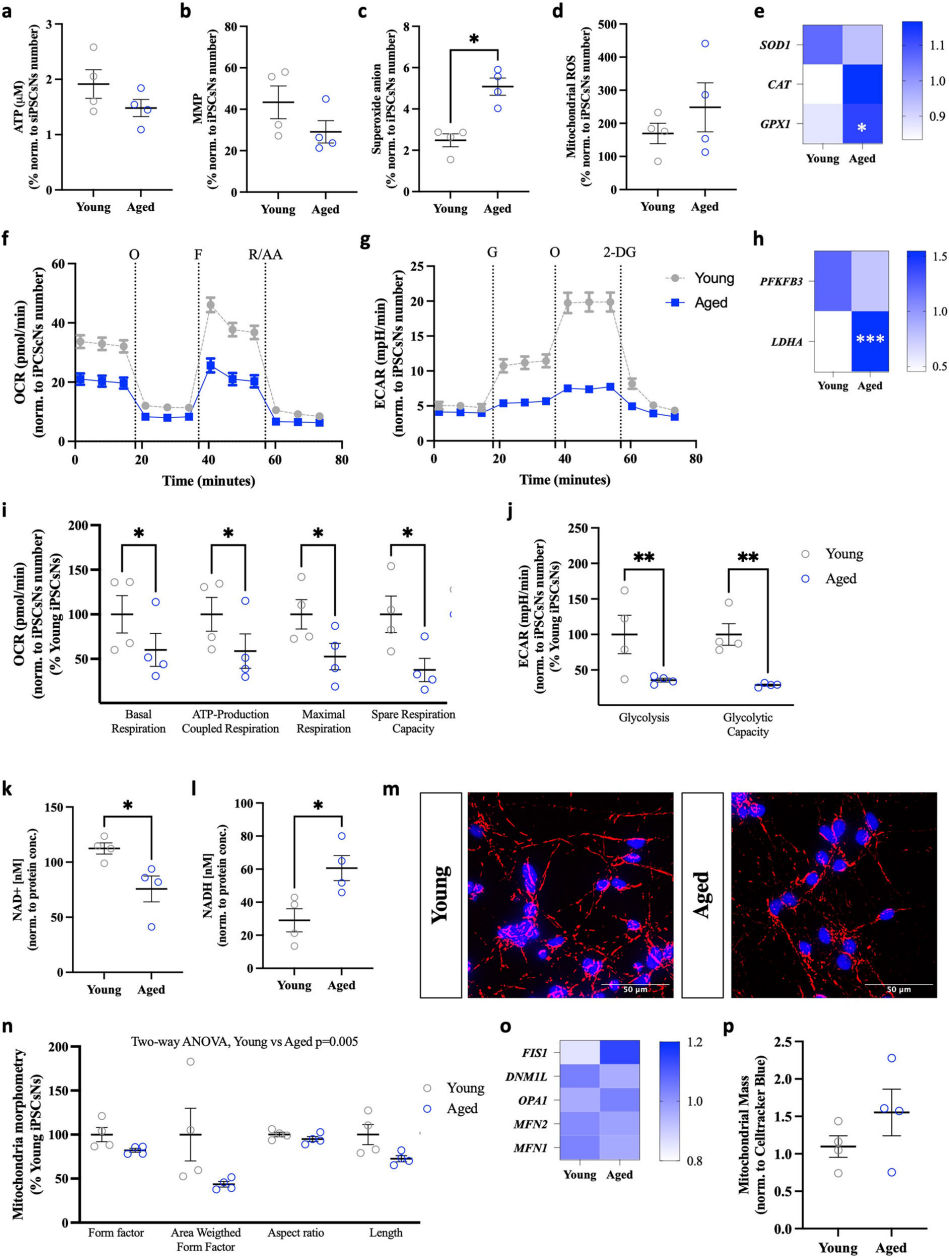
A decrease in mitochondrial function was seen in aged iPSCsNs compared to young iPSCsNs, but no evidence of a metabolic shift towards anaerobic glycolysis. **a** Cellular ATP level comparing iPSCsNs from aged and young human donors (*N* = 5 independent experiments, *n* = 2–3 replicates per experiment for each donor). **b** MMP level was measured in aged iPSCsNs and young iPSCsNs by staining with TMRM. The fluorescence was detected at ex: 548 nm/em: 574 nm (*N* = 5 independent experiments, *n* = 2–3 replicates per experiment for each donor). **c** Mitochondrial superoxide anion detection using the MitoSOX dye to compare young and aged iPSCsNs. The fluorescence was detected at ex: 485 nm/em: 535 nm (*N* = 5 independent experiments, *n* = 3 replicates per experiment for each donor). **d** Mitochondrial ROS detection in aged and young iPSCsNs. (ex: 485 nm/em: 535 nm). The fluorescence was detected at ex: 531 nm/em: 595 nm (*N* = 5 independent experiments, *n* = 3 replicates per experiment for each donor). **e** Relative gene expression of relevant anti-oxidative stress enzymes, *SOD1, CAT*, and *GPX1*. The data are represented as gene expression (2 ^(−Avg.(Delta(Ct))^) as the Housekeeping gene GAPDH was utilized. **f** Mito Stress Test profile representing the OCR of young and aged iPSCsNs after sequential injection of oligomycin (O, 2,5 µM), FCCP (F, 2 µM), and lastly combined rotenone (R, 2 µM) with antimycin A (A, 2 µM). **g** Glycolysis Stress Test profile representing the ECAR of aged and young iPSCsNs after sequential injection of glucose (G, 10 mM), Oligomycin (O, 1 µM), and lastly 2-deoxy-glucose (2-DG, 25 mM). **h** Bioenergetic parameters of the mitochondria of young and aged iPSCsNs. Basal respiration, ATP-production coupled respiration, maximal respiration, and spare respiration capacity (*N* = 4 independent experiments, *n* = 2–3 replicates per experiment for each donor). **i** Bioenergetic parameters of glycolysis comparing young and aged iPSCsNs. Glycolysis and glycolytic capacity (*N* = 3 independent experiments, *n* = 2–3 replicates per experiment for each donor). **j** Relative gene expression of relevant glycolysis-regulating genes, *PFKFB3, PKM, LDHA*. The data are represented as gene expression (2 ^(−Avg.(Delta(Ct))^) as the Housekeeping gene GAPDH was utilized. Cellular NAD^+^ content (**k**) and NADH content (**l**) from young and aged iPSCsNs represented as normalized values to the protein concentration (*N* = 5 independent experiments, *n* = 3 replicates per experiment for each donor). **m**, **n** Mitochondrial network morphology (**m**) was assessed in iPSCsNs from young and aged human donors by visualizing the mitochondria with TOMM20 and nucleus staining with DAPI. Calculated mitochondrial parameters (**n**) Form Factor, Area Weighted Form Factor, Aspect Ratio, and Length (*N* = 5 independent experiments, *n* = 3 replicates per experiment for each donor). **o** Relative gene expression of relevant genes involved in mitochondrial dynamics: *FIS1, DNM1L, OPA1, MFN2*, and *MFN1*. The data are represented as gene expression (2 ^(−Avg.(Delta(Ct))^) as the Housekeeping gene GAPDH was utilized. **p** Mitochondrial Mass comparing young and aged iPSCsNs assessed by using the MitoTracker™ Green FM (ex: 490 nm/em: 516 nm) to stain mitochondria and were normalized to the cell area using Celltracker blue (ex: 353 nm/em: 466 nm) (*N* = 5 independent experiments, *n* = 3 replicates per experiment for each donor). Data information: All data are represented as the mean ± SEM of each four different young and aged iNs. Statistical parameters, including the number of values, minimum, maximum, range, mean, standard deviation, and standard error of the mean, are presented in Supplementary Data 2. Only three donors were assessed for gene expression with three technical replicates. Values were normalized on the cell count. Non-parametric Mann–Whitney test was performed to compare young iPSCsNs versus aged iPSCsNs (*p < 0.05, **p < 0.01, ***p < 0.001), or a two-way ANOVA was applied to compare multiple parameters.

Consistent with our observations in HFs and iNs, cellular ATP levels exhibited a decreasing trend in aged iPSCsNs when comparing the four young donors to the four aged donors (Fig. 3a). When analyzing individual data points, a significant reduction in ATP levels was evident in aged iPSCsNs (Fig. S3a). Furthermore, we observed a downward trend in MMP (Fig. 3b), which was statistically significant when comparing individual data points (Fig. S3b). The superoxide anion radical produced by mitochondria (Fig. 3c) and the mitochondrial ROS (Fig. 3d) rose in aged iPSCsNs, with the rise in mitochondrial ROS reaching statistical significance when comparing the individual young donors versus aged donors. Further investigating the gene expression of antioxidant defense proteins (Fig. 3e), we only found a significant increase in *GPX1* mRNA expression in aged iPSCsNs. During our investigation, we monitored the mitochondrial respiration (Fig. 3f, i) to discover a significant decrease in aged iPSCsNs. After conducting the Seahorse Glycolysis Stress Test (Fig. 3g), we identified a substantial decline in the glycolysis parameter (Fig. 3j) in the aged iPSCsNs. The gene expression analysis of critical genes in glycolysis revealed that *PFKF3B* remained unchanged. Nevertheless, we observed that the *LDHA* mRNA expression in aged iPSCsNs was significantly evaluated than in young iPSCsNs. Upon evaluating the NAD^+^ to NADH ratio, we observed a substantial decrease in the NAD^+^ level (Fig. 3k) and a significant increase in the NADH level (Fig. 3l) in aged iPSCsNs. The NAD^+^/ NADH ratio (Supplementary Data 1) was 3.87 in young iPSCsNs. In contrast, the ratio was 1.25 in aged iPSCsNs, indicating a substantial drop in the NAD^+^/NADH redox ratio. While examining the mitochondrial morphology in iPSCsNs (Fig. 3m, n), we observed that the aged iPSCsNs exhibited a more fragmented mitochondrial network morphology, as evidenced by a decline in FF, AW, AR, and mitochondria length. Mitochondrial morphometry analysis (Fig. 3n), evaluated using two-way ANOVA, revealed a statistically significant difference between the young and aged groups (*p* = 0.005). Our study showed that the mitochondrial mass (Fig. 3p) was higher in aged iPSCsNs than in young iPSCsNs. However, no significant changes were found in the mRNA expression of mitochondrial dynamics genes between young and aged iPSCsNs.

### Transcriptomic alteration in the neuronal models of aging

**Fig. 4:**
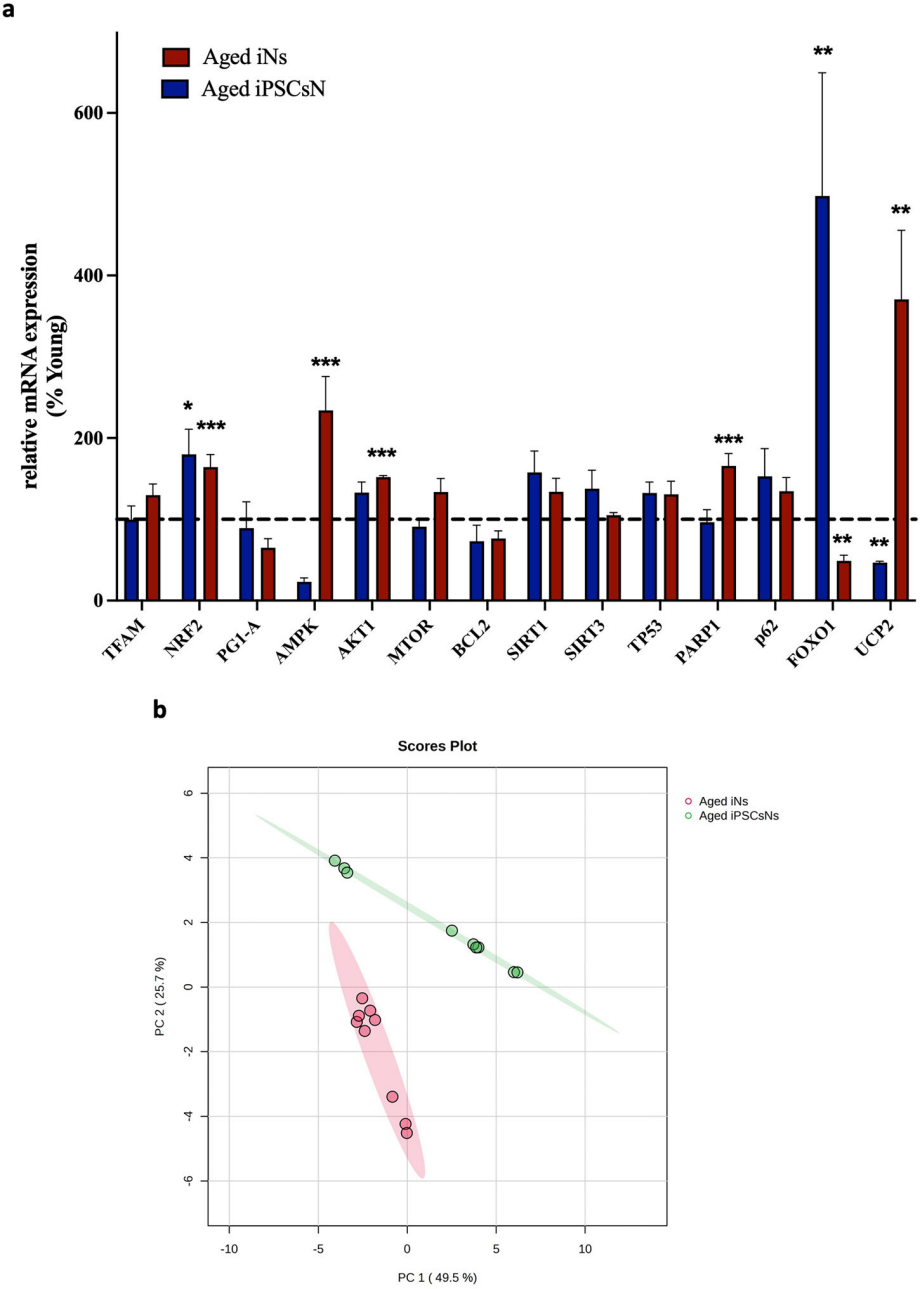
Genes related to mitochondrial properties show transcriptomic alterations in neuronal models of aging. **a** The mRNA expression profile of aged iNs and aged iPSCsNs % normalized to the corresponding young. The smaller graph represented the mRNA expression of the genes *FOXO1* and *UCP2*, which had the highest percentage difference compared to the corresponding young (*N* = 3 independent experiments, *n* = 3 replicates per experiment). **b** Aged iNs versus aged iPSCsNs of detected genes in our investigation. Principal component analysis of aged iNs (red) and aged iPSCsNs (green). Scores plot between the selected PC1 and PC2. Each collared dot represents a replicate (*N* = 3 independent experiments, *n* = 3 replicates per experiment). Data information: The represented values for the gene expression were assessed from three donors with three technical replicates for each condition. Student’s unpaired t-test was performed to compare the gene expression between young and aged iNs and between young and aged iPSCsNs. Therefore, the asterisks (*p < 0.05, **p < 0.01, ***p < 0.001) indicate a statistically significant difference between aged iNs vs. young iNs or young iPSCsNs vs. aged iPSCsNs, analyzing the same neuronal models between young and aged groups (*p < 0.05, **p < 0.01, ***p < 0.001). The detailed statistical parameters, including the number of values, minimum, maximum, range, mean, standard deviation, and standard error of the mean, are presented in Supplementary Data 2. **b** was generated on metaboanalyst.ca. The represented values were normalized by autoscaling (mean-centered and divided by the standard deviation of each variable). The data were represented as Gene expression (2^(−Avg.(Delta(Ct))^) by using the housekeeping gene *GAPDH*, which is calculated by the website https://geneglobe.qiagen.com/us/analyze. **b** was generated using metaboanalyst.ca.

Succeeding, we investigated the mRNA expression of further mitochondrial-associated crucial genes (Fig. 4a). Our investigation revealed notable alterations in the mRNA expression of poly (ADP-ribose)-polymerase-1 (PARP1), Serine/threonine-protein kinases 1 (AKT1), AMP-activated protein kinase (AMPK), Nuclear factor erythroid-derived 2-like 2 (NRF2), Forkhead box protein O1 (FOXO1), and uncoupling protein 2 (UCP2) in aged iNs when compared to young iNs. The mRNA expression of NRF2, FOXO1, and UCP2 in aged iPSCsNs differed significantly from that in young iPSCsNs. Nevertheless, the remaining genes exhibited no alteration. Along with NRF2, peroxisome proliferator-activated receptor gamma coactivator 1 alpha (PGC-1α) triggers mitochondrial biogenesis (mitogenesis)^29,30^. Regarding the gene regulation of mitogenesis, we found that only the *NRF2* gene showed a notable increase in expression in both aged neuronal models. Upon examining the upstream regulator of PGC-1α, we discovered that *FOXO1*, was significantly downregulated in aged iNs and upregulated in aged iPSCs than the corresponding young. Another upstream regulator, AMPK, was elevated in aged iNs, but no significant disparities in AMPK gene expression were observed in the iPSCsNs-state^30^. Further investigation was conducted into the inhibitory pathways of PGC-1α, including AKT1^30^. For the *AKT1* mRNA expression, only aged iNs showed a significant upregulation than young iNs, while there was no discernible difference in the iPSCsNs. Other genes that showed a significant upregulation in the aged iNs, as reflected in aging^31^ but not in the aged iPSCsNs were *PARP1* and *UCP2*. Given the pronounced changes between both neuronal models, we investigated whether there would be differences in the mRNA expression by directly comparing the aged neurons. Therefore, we performed a principal component analysis (PCA) comparing the gene expression profile of all detected genes between the aged neuronal models. Using the first two principal components, we visually identified that the aged iNs and aged iPSCsNs data sets did not cluster (Fig. 4b).

### Comparative analysis of mitochondrial parameters

We conducted a comparative analysis of the critical mitochondrial parameters to understand how the conversions affect the mitochondrial properties at the individual donor level. In a spiderweb comparison (Figs. S5 and S6) of the various cell models, for each donor. We noticed a significant alignment in distinct characteristics during the transformation process. However, there were variations in specific characteristics among the individual donors. In the young donors (Fig. S5), we noticed differences in the maximal respiration capacities among the various cell types. Specifically, when comparing Young 2 and Young 3, we found that Young 2 had the highest maximal respiratory capacity among the iNs. In contrast, Young 3 experienced a significant decline in the iNs’ state of maximal capacity. Interestingly, the other cell states, HFs and iPSCsNs, did not exhibit such differences. We detected a substantial rise in the mitochondrial ROS parameters and NAD^+^ levels in the iNs state of Young 3 compared to the HFs and iPSCsNs state and showing the most substantial variation between the young donors in the iNs state, while the HFs’ and iPSCsNs’ states appeared more like the other donors. In the comparison between the different aged donors (Fig. S6), it was observed that aged donors in different reprogramming states shared more similarities than young donors. Moreover, we did not observe any difference between the male donor (Aged 1) and the female donors (Aged 2–4). Nevertheless, Aged 3 (female) showed in the HFs state a lower ROS emission compared to the other aged donors.

### Telomere alteration

**Fig. 5:**
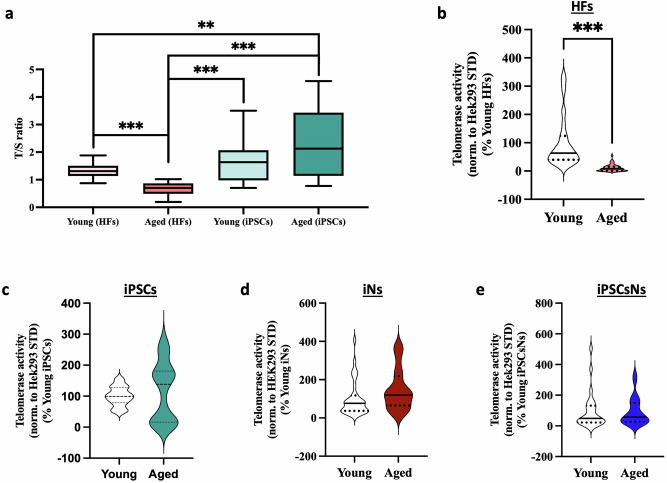
Telomere impairments in aged HFs but not in the derived cell types. Telomere length measurement (**a**) of young and aged HFs and young and aged iPSCs. The data are represented as the TELO/ SGC (T/S). A mixture of all HFs’ DNA samples was used as an internal control (*N* = 4–5 independent experiments, *n* = 3 replicates per experiment). The telomerase activity from young and aged donors was measured in HFs (**b**), iPSCs (**c**), iNs (**d**), and iPSCsNs (**e**). The assessment was done in 10,000 cells for HFs and iPSCs and 100,000 cells for both neuronal models. The data are represented as values normalized to a Hek293 STD and normalized to 100% of the corresponding young (*N* = 2–6 independent experiments, *n* = 1–2 replicates per experiment). Data information: All data are represented as the mean ± SEM of each four different young and aged HFs, iPSCs, iNs, or iPSCsNs. Except for T/S iPSCs, only three donors were utilized. Statistical parameters, including the number of values, minimum, maximum, range, mean, standard deviation, and standard error of the mean, are presented in Supplementary Data 2. The represented values from four young and aged donors derived iNs show *N* independent experiments with n technical replicates per donor. Student’s unpaired *t*-test was performed for young versus aged (*p < 0.05, **p < 0.01, ***p < 0.001).

Our analysis further concentrated on telomere length (TL) shortening as a known aging hallmark, particularly of proliferating cells^32^. Our study showed TL decreased with age in HFs but remained unchanged in the iPSCs state (Fig. 5a). Interestingly, we also observed an increase in TL from HFs to iPSCs, with young donors increasing from 1.34 T/S to 1.678 T/S and aged donors increasing from 0.66 T/S to 2.32 T/S after conversion. We investigated the telomerase activity (TA), which counteracts the telomere attrition in healthy cells^33–36^. We detected a significant decrease in TA in aged HFs compared to young HFs (Fig. 5b). However, no significant differences were found between young and aged iPSCs (Fig. 5c), iNs (Fig. 5d), and iPSCsNs (Fig. 5e).

## Discussion

**Fig. 6:**
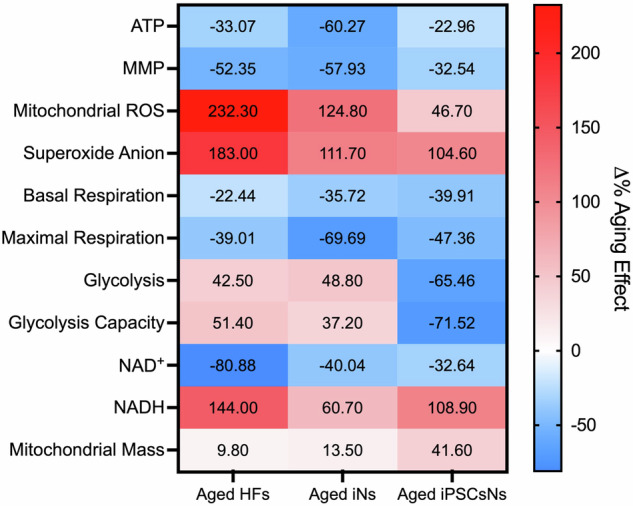
Δ% Aging effect in mitochondrial key parameters. The data are represented as a heatmap comparing the aging effect across HFs, iNs, and iPSCsNs. The heatmap represents the Δ% aging effect relative to the corresponding young samples, based on individual data points.

In this comparative study, iNs and iPSCsNs, derived from the same donor HFs, were evaluated to identify the most appropriate model for investigating neuronal aging in vitro. Our research uncovered substantial variations in the mitochondrial bioenergetic status between young and aged HFs and iNs. Importantly, these differences were also observed in the iPSCsNs, a surprising outcome not previously reported^18,37–39^. Generally, aged iNs displayed closer similarities to the original cells than aged iPSCsNs (Fig. 6). However, some exceptions were noted, such as the pronounced aging-related impact on NADH levels and basal respiration in aged iPSCsNs. Notably, a significant disparity was the absence of a metabolic shift towards glycolysis in aged iPSCsNs, a phenomenon observed in HFs and iNs from aged individuals. The extent to which reprogramming iPSCs and the resulting cells can effectively reset the metabolic and cellular alterations associated with aging remains a subject of ongoing debate. While some studies suggest an overall rejuvenation, others indicate that aging-associated impairment could be preserved to some extent^24^. Our previous study^26^ demonstrated an aging-associated impairment in the aged iPSCs compared to young iPSCs, which we could replicate in the neuronal state. Our findings indicate that aged iPSCsNs exhibit similar impairments in distinct mitochondrial properties as aged iNs.

Our findings reveal that whether directly converted or iPSC-derived, aged neurons exhibit energy impairments, particularly in mitochondrial function, aligning with previous studies on neuronal aging^40–45^. Specifically, these aged neuronal models show reduced energy production due to a decline in mitochondrial respiratory chain activity, reinforcing the oxidative stress theory of aging, which posits that free radical damage to cellular components accelerates the aging process^46–48^. This is evident by the increased concentration of free radicals in our aged neuronal models, predominantly generated through NAD-related energy pathways^49,50^. A high NAD^+^/NADH ratio is a hallmark of healthy cellular and mitochondrial function, while a decrease signals impairments^51,52^. Our findings in aged neuronal models show a reduction in NAD^+^ and an increase in NADH with aging, resulting in a lower NAD^+^/NADH ratio, consistent with previous studies^40,53–56^. Further, the mitochondrial network evaluation reveals additional insights into the bioenergetic state and overall neuronal health^57–59^. Proper mitochondrial distribution is essential for fulfilling the neuronal energy requirements^60^. As we age, our cells tend to accumulate more dysfunctional mitochondria due to diminishing mitophagy processes hampered by oxidative stress^30^, as highlighted in our neuronal models. In response to mitochondrial impairments, cells shift to a more glycolytic metabolism, which still fails to resolve energy deficits, leading to higher LDHA levels and potential lactate toxicity with age^61–64^. Consistent with previous studies, our findings show increased glycolytic activity in aged HFs and iNs, indicating the attempt to maintain a consistent energy level in aging^65,66^. The observed reduction in ATP levels in aged iNs, induced adequately by the mitochondrial impairments despite the significant increase in ECAR, highlights a critical bioenergetic imbalance. Under normal conditions, neurons rely heavily on mitochondrial OXPHOS for efficient ATP production, with glycolysis being supportive^67,68^. However, in age, mitochondrial bioenergetic impairments force the cells to upregulate glycolysis as a compensatory mechanism. While glycolysis generates ATP rapidly, it is far less efficient than OXPHOS, producing only two ATP molecules per glucose molecule compared to the significantly higher ATP yield of ~30 ATP^69^. The upregulation of glycolysis alone may not suffice to meet the cell’s energy demands, explaining the significant reduction in ATP content. The mitochondrial impairment in aged iNs might be so severe that even increased glycolysis cannot fully compensate for energy loss by the mitochondria^2^. Moreover, elevated ECAR might also indicate lactate accumulation rather than effective ATP synthesis, suggesting that glycolytic flux is diverted toward lactate production rather than feeding into the TCA cycle^70^. This could be further compounded by the potential rerouting of glycolytic intermediates into the pentose phosphate pathway (PPP) for NADPH production or the serine synthesis pathway (SSP) for producing serine, a critical mechanism for combating oxidative stress in aged cells^71,72^. Additionally, aged iNs may face increased energy demands due to persistent cellular stressors such as disrupted calcium homeostasis, oxidative damage, and protein misfolding linked to aging, which could further deplete ATP reserves despite enhanced glycolysis^73,74^. The severe bioenergetic crisis observed suggests that mitochondrial impairment in aged iNs surpasses the compensatory capacity of glycolysis, ultimately leading to energy failure. Unlike aged iNs, iPSCsNs did not show an aging-related metabolic shift in real-time, but we observed a significant increase in LDHA expression, contradicting the real-time assessment. The mitochondrial impairments in our aged iPSCsNs might cause a corresponding rise in the *LDHA* expression. Nevertheless, as iPSCsNs undergo various reprogramming steps, their ability to adapt to the aging-associated energy deficit could be reset. Although mitochondrial dysfunction can affect the transcriptomic level of aged iPSCsNs, it does not similarly impact their glycolytic behavior. The absence of discernible differences in the mRNA expression of the *PFKFB3* gene between iPSCsNs from young and aged individuals supports our speculation. This suggests a diminished ability to shift to glycolytic metabolism, possibly due to partial rejuvenation. Moreover, previous research suggests that inducing pluripotency resets age-related gene expression, while direct conversion retains aging-associated donor signatures^12,22^. It is important to understand that some glycolytic enzymes are primarily regulated by allosteric mechanisms and post-translational modifications rather than solely through changes in mRNA expression. For instance, the ATP-dependent phosphorylation of fructose-6-phosphate to form fructose 1,6-bisphosphate is catalyzed by phosphofructokinase-1 (PFK1)^75–78^. This reaction is a crucial step in glycolysis and serves as a key control point for regulating glycolytic flux, making PFK1 an essential regulator of this metabolic pathway. PFK1 is influenced by the allosteric activator fructose-2,6-bisphosphate, which PFKFB3 regulates. Interestingly, PFKFB3 itself is continuously degraded in healthy neurons by the E3 ubiquitin ligase anaphase-promoting complex and its coactivator, cadherin 1 (Cdh1). Nevertheless, the downregulation of glycolysis could indicate a compensatory upregulation of the PPP, enhancing oxidative stress resilience^71^. Given our observation of mitochondrial impairments, we anticipated similar transcriptomic profiles between aged iNs and aged iPSCsNs with the same genetic background, but our findings revealed distinct differences. Aged iNs showed more disparities compared to young iNs, indicating that direct conversion preserves functional impairments, whereas reprogramming via iPSCs state induced a partial rejuvenation.

Recent studies have linked mitochondrial malfunction to the progressive shortening of telomeres (TL), a key hallmark of aging^79,80^, as observed in our source cell. While we found no significant differences in TL and TA between young and aged iPSCs, TL was notably prolonged in iPSCs compared to their donor cells, suggesting a rejuvenation effect^18,81,82^. Similarly, no TA differences were detected in the neuronal models. Unlike dividing cells, neurons may not require TA, though research shows that telomerase proteins are produced in post-mitotic neurons, albeit at a lower rate^34,83,84^. This could also be due to alterations occurring during the conversion process, raising the questions about how other aging hallmarks are affected, requiring further investigation into the preservation of aging characteristics during the reprogramming process.

When examining changes linked to human brain aging, it is essential to consider aging plasticity^85^. We selected healthy aged donors (60+ years), a group at higher risk for age-related disorders^86–88^, and compared them to healthy young adults (24-36 years) representing a fully maturated brain^89^. Our analysis revealed that aging can vary among individuals and cellular models. The results highlight that donor-specific traits persist in our neuron models after reprogramming, with more pronounced differences among young individuals than aged donors^90^. These findings underscore the significance of comprehending the adaptability of aging and its unique impact on mitochondrial properties, suggesting that comparing two individuals alone may not fully capture a pathological phenotype.

Contrary to previous assumptions^19,20^, our research reveals that aged iPSCsNs can retain their donor-associated aging signature at the mitochondrial level. While aged iPSCsNs show promise as neuronal models for mitochondrial aging, they fail to replicate the mRNA and glycolysis phenotypes of aged iNs, suggesting a partial rejuvenation mechanism. These findings highlight the need for further research into the distinct aging characteristics of iPSCsNs and iNs to fully understand their potential as models for studying age-related neuronal changes.

## Conclusion

Our findings in this preliminary investigation indicate that aged iNs and iPSCsNs from the *same aged cells of origin* exhibit a wide array of mitochondrial aging phenotypes akin to those observed in in vivo aging. Particularly, aged iNs represent great potential as neuronal models representing an overall aging phenotype. Nevertheless, using iNs carries technical limitations, especially regarding neuron yield^38,91^. Due to the low conversion rate of aged iNs, we were unable to gender-match our study. The limited number of successfully converted neurons restricted our ability to include equal representations of male and female samples. While we cannot entirely exclude gender differences, it is unlikely to have a significant impact, as no mitochondrial variations were observed between genders (Supplementary Fig. 3). This is consistent with previous research showing no gender differences in mitochondrial respiration, ATP levels, or oxidative stress in mice^92^ and no differences in iPSCs studies^93^. Despite the gender imbalance, the insights gained from their study remain robust and valuable. As mitochondrial function is a central focus of this study, the lack of observed gender-based differences supports the robustness of our findings. However, future studies should explore potential sex-specific influences beyond mitochondrial parameters to ensure a comprehensive understanding of neuronal aging. While most studies focus on age-related neurodegenerative diseases, there is a gap in understanding molecular changes in the aging brain without disease. Jiao and colleagues conducted similar work, comparing iPSCsNs and iNs from Dravet syndrome patients, showing that both models represent a hyperexcitable state linked to the pathological phenotype^94^. Research on iPSCsNs from Alzheimer’s patients also revealed high levels of AD-related markers^95–97^, though more extended cultivation may be needed to study AD-associated changes. However, studies demonstrating preservation of aging or pathological phenotypes in iPSCs or iPSCsNs remain rare.

**Fig. 7:**
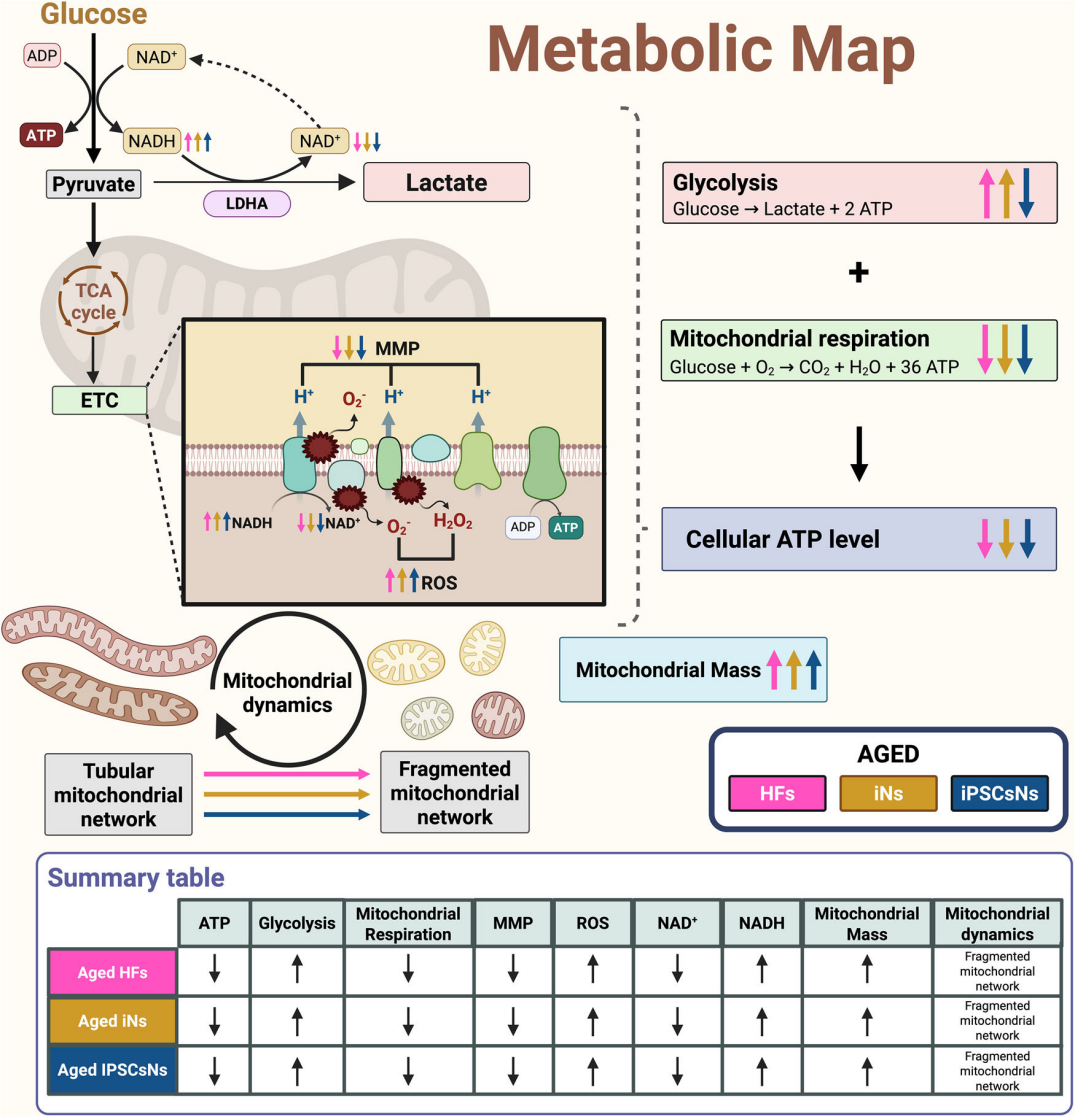
Schematic representation of the metabolic pathways in our neuronal models of aging. This metabolic map illustrates the interconnected energy metabolism pathways in our neuronal aging models (iNs and iPSCsNs) compared to their donor’s cells (HFs), emphasizing key processes involved in mitochondrial properties and glycolysis. The arrow depicts the directionality of the aging effect in the different cellular models, whereby ↑-arrow indicates a rise and ↓-arrow indicates a decline in age compared to the young counterparts. The color-coded arrows differentiate between HFs (pink), iNs (ocker), and iPSCsNs (blue). Created in BioRender. Grimm, A. (2025) https://BioRender.com/3v5ky5y.

Both neuronal models represent important model systems for investigating neuronal aging as an ethical alternative to animal models^98^. However, there is a need for more research examining the maintenance of the aging characteristics in iPSCsNs and iNs. Especially to understand human-specific neuronal connectivity, modeling neuronal disorders, and aiding drug discoveries underlying neurodegenerative disorders. As these neuronal models can be generated from every single individual, personalized medicine can be performed to target an individual’s needs. Both neuronal models represent the capacity of action potential, form synaptic transmission, electrophysiological properties, and neuronal transmitter release^99,100^. However, the two model systems differ based on current understanding^13,99,101,102^. iNs are considered to mature faster and offer subtype specificity, while iPSCsNs demonstrate a higher level of synaptic plasticity. Although iNs are considered to develop action potentials more rapidly, iPSC-derived neurons may achieve complete maturation, allowing for a broader range of functional and subtype-specific properties. In our study, we focused on investigating the overall neuronal cultures in our experiments, whereby examining how aging phenotypes manifest at the level of individual cells was neglected. This represents a potential area for further exploration, particularly in understanding how these aging phenotypes are maintained or altered in individual cells and whether they are heritably transmitted to daughter cell lines during division. Given that both neuronal models originate from dividing cells, understanding these dynamics could provide critical insights into the stability and progression of aging-related cellular characteristics. Despite certain constraints, our findings offer valuable insights and serve as a preliminary investigation into potential models of human neuronal aging. While this study lays the groundwork, further validation of additional aging parameters, especially those related to other mitochondrial properties (not explored here) and broader cellular mechanisms, is crucial for achieving a more comprehensive understanding. In summary, our work deepened our understanding of how accurate iNs or iPSCsNs derived from aged donors accurately replicate brain aging. Aged iPSCsNs display a mitochondrial-associated aging phenotype akin to aged iNs, challenging the notion of a comprehensive rejuvenation process in iPSCs and their derived cells (Fig. 7). This highlights aged iPSCsNs as a valuable neuronal in vitro model due to their widespread availability and retention of aging-related mitochondrial deficits, besides aged iNs.

## Material and methods

### Reagents and tools

All reagents and tools used in this study are listed in Table 1.

**Table 1:** Reagents and tools

Reagent/Resource	Reference or source	Identifier or catalog number
Experimental models		
HFs	Takara Bio and The Cader Laboratory	Please see the representative table
iPSCs	Takara Bio and The Cader Laboratory	Please see the representative table
HEK293	The Eckert Laboratory	N/A
Antibodies		
Recombinant Alexa Fluor® 555 Anti-TOMM20 antibody	Abcam	ab221292
Anti-PSA-NCAM-PE, human, mouse, rat	Milteny Biotec	130-117-394
Anti-MAP2 antibody	Abcam	ab32454
Neuron-Specific Beta-III Tubulin Antibody	R&D Systems	MAB1195
Oligonucleotide		
Primers for gene expression	Qiagen	Please refer to the corresponding table
Primers TL	Microsynth AG	Custom-made (please refer to the corresponding table)
pLVX-UbC-rtTA-Ngn2:2A:ASCL1 (UNA)	Salt institute (CA, USA) (now available on Addgene)	#127289
Chemicals, peptides, enzymes, and other reagents		
Seahorse XF DMEM Medium, pH 7.4	Agilent Technologies	103575-10
2-deoxy-glucose	Sigma Aldrich	D6134-5G
2-mercaptoethanol	Sigma Aldrich	#63689
3-(4,5-Dimethylthiazol-2-yl)-2,5-diphenyltetrazolium bromide (MTT)	Sigma Aldrich	M2128
4-(2-Aminoethyl)benzolsulfonylfluorid -hydrochlorid (AEBSF)	Sigma Aldrich	A8456
4′,6-Diamidin-2-phenylindol (DAPI)	Sigma-Aldrich	D9542-50MG
A83-1	Santa Cruz Biotechnology, Inc.	SC-203791
Accutase	Innovative Cell Technology	ICTAT104
Active Recombinant Human Noggin/NOG Protein	Abclonal Technology	10267-HNAH
Advanced DMEM/F-12	Gibco	#12634028
Alcohol dehydrogenase	Sigma Aldrich	A3263-15KU
Antimycin A	Sigma Aldrich	A8674
AZ960	AdipoGen Life Science	AG-CR1-3752
B-27 Supplement (50X)	Gibco	#17504001
Bovine serum albumin (BSA)	Sigma Aldrich	A9647
BrainPhys	STEMCELL Technology	#05790
Carbonyl cyanide-p-trifluoromethoxyphenylhydrazone (FCCP)	Sigma Aldrich	C2920
Cellartis DEF-CS 500 COAT-1	Takara Bio	Y30012
Cellartis DEF-CS 500 Culture System	Takara Bio	Y30010
Cellartis DEF-CS additives GF-1, GF-2, GF-3	Takara Bio	Y30016
CellTracker™ Blue CMAC Dye	Invitrogen	C2110
CHIR-99021	LC-Laboratories	C-6556
DAPI	Merck	10236276001
DB Puromycin 2 HCI (Powder)	BioConcept Ltd. Amime	4-17P00-DB
db-cAMP	MedChemExpress	HY-B0764/CS-2967
Dihydrorhodamine 123 (DHR)	Thermo Fisher Scientific	D1054-10mg
DMEM/F-12, no glutamine	Gibco	#21331020
Doxycycline hyclate	Sigma Aldrich	D9891
Dulbecco’s modified Eagle’s medium (DMEM)	Invitrogen	D6429
EDTA	Sigma Aldrich	E6758
Ethanol	Sigma Aldrich	#02860
Formaldehyde	Sigma Aldrich	F8775
Forskolin	LC-Laboratories	F-9929
Geltrex LDEV-Free hESC-Qualified Reduced Growth Factor Basement Membrane Matrix	Gibco	A1413302
Gibco™HBSS, calcium, magnesium, no phenol red	Gibco	#14175050
GlutaMax	Gibco	#35050061
Glutamine	Agilent Technologies	#103579-100
Glycerol	Sigma Aldrich	G5516
HCl	Carl Roth GmbH	X896.1
Horse serum (HS)	Capricon	HOS-1A
Human GDNF	PrepoTech	450-10-10ug
Isopropanol	Merck	#1027811000
KC7F2	APExBIO	A4507
Laminin	Roche	#11243217001
LDN-193189	Cayman	CAY-11802-10
MEM Non-Essential Amino Acids Solution (100X) (NEAA)	Gibco	#11140050
MgCl_2_(6 H_2_O)	Sigma Aldrich	M2670
MitoSOX™ Red Mitochondrial Superoxide Indicator	Thermo Fisher Scientific	M36008
MitoTracker™ Green FM	Thermo Fisher Scientific	M7514
Myo-inositol	Sigma Aldrich	I5125
N-2 Supplement (100X)	Gibco	#17502048
NaCl	Merck	#1.06404.1000
NaOH	Fluka Analytical	#30531
Neurobasal Medium minus phenol red	Gibco	#12348017
Neurobasal™-A Medium	Gibco	#10888022
NP-40	Roche diagnostics	#1754599
NucBlue™ Fixed Cell ReadyProbes™ Reagent (DAPI)	Invitrogen	R37606
Oligomycin	Sigma Aldrich	O4876
PBS	Dominique Dutscher	X0520-500
Penicillin/Streptomycin	Bioconcept	4-01F00-5
Phenazine ethosulfate (PES)	Sigma Aldrich	P4544
Polyvinyl alcohol	Sigma Aldrich	P8136
PSC Neural Induction Medium	Gibco	A1647801
Pyrintegrin	Tocris	#4978
Recombinant Human EGF	Peprotech	AF-100-15
Recombinant Human FGF-basic	Peprotech	100-18C
Recombinant Human/Murine/Rat BDNF	Peprotech	450-02
RHB-A	Takara Bio	Y40001
RHB-BASAL	Takara Bio	Y40000
Rotenone	Sigma Aldrich	#45656
RQ1 RNase-Free DNase	Promega	M6101
SB-431542	MedChemExpress	HY-10431-50MG 5
Seahorse XF 100 mM Pyruvate Solution	Agilent Technologies	103578-100
Seahorse XF 1 M Glucose Solution	Agilent Technologies	103577-100
Sodium deoxycholate	Sigma Aldrich	#30970
StemPro Accutase Cell Dissociation Reagent	Gibco	A1110501
Tetracycline-free fetal bovine serum (FBS)	Gibco	A5256701
Tetramethyl rhodamine, methyl ester, and perchlorate (TMRM);	Sigma Aldrich	T5428
Tris	Pharmacia Biotech	17-1321-01
Tris-HCl	Carl Roth GmbH	#9090.3
Triton X-100	Sigma Aldrich	T8787
TrypLE™ Select Enzyme	Thermo Fisher Scientific	#12605010
Vectashield H-1000 mounting medium	Vector	H-1000
Y-27632 (ROCK Inhibitor)	Selleck Chemicals	S6390
z-VAD-FMK	Enzo Life Science	ALX-260-020-M001
ZM 33637	Cayman	#10010367
Critical commercial assays		
ATPlite 1step Luminescence Assay System	Perkin Elmer	#6016739
Custom RT^2^ Profiler PCR Arrays	Qiagen	#330231
DC™ Protein Assay Kit II	Bio Rad	#5000112
FlexiGene® DNA KIT	Qiagen	#51206
GoTaq® Probe qPCR Master Mix	Promega	A6102
MycoAlert® PLUS Mycoplasma Detection Kit	Lonza	LT07-701
RNeasy Mini Kit	Qiagen	74106
RT^2^ First Strand Kit	Qiagen	#330401
RT^2^ SYBR Green ROX qPCR Master Mix	Qiagen	#330513
Software and algorithms		
BioRender	BioRender	BioRender.com
Excel software	Microsoft Corporation	N/A
Fiji (Fiji is just ImageJ)	NIH	https://imagej.net/software/fiji/
Gen5 Image 3.11 software	Agilent Technologies	N/A
GeneGlobe Data Analysis Centre Software	Qiagen	https://geneglobe.qiagen.com/us/analyze
Huygens Deconvolution Software	Scientific Volume Imaging	https://svi.nl/Huygens-Software
MetaboAnalyst 5.0	MetaboAnalyst	https://www.metaboanalyst.ca/home.xhtml
Prism version 10.1.1	GraphPad	https://www.graphpad.com/scientific-software/prism/
R-software	R Foundation	https://www.r-project.org
SABiosciences PCR Array Data Analysis Software	Applied Biosystems	N/A
Software Nikon NIS-HC	Nikon Instruments	NIS-Elements HC

#### Cell culture

The primary HFs and the corresponding iPSCs were purchased from Takara Bio (Kusatsu, Shiga, Japan) or were kindly provided by Dr. Zameel Cader (University of Oxford) and the Stem Cells for Biological Assays of Novel Drugs and Predictive Toxicology (StemBANCC) consortium. All HFs or iPSCs in this study are either commercially available or obtained from biorepositories. Therefore, they are exempt from the Human Research Act and do not require ethical approval. Furthermore, none of the donors were diagnosed with any diseases at the time of the biopsy, which means all donors were considered healthy. For an in-depth comprehension of donor information, please see Table 2.

**Table 2:** Donor information of the HFs and iPSCs

Category	Internal labeling	Identifier or catalog number (HFs)	Identifier or catalog number (iPSCs)	Age	Gender	Source
Young	Y1	Cellartis® Fibroblast P11031-C12	Cellartis® Human iPS Cell Line 12	24 Y	M	Takara Bio
	Y2	Cellartis® Fibroblast P11019-C18	Cellartis® Human iPS Cell Line 18	32 Y	M	Takara Bio
	Y3	Cellartis® Fibroblast P11028-C22	Cellartis® Human iPS Cell Line 22	32 Y	M	Takara Bio
	Y4	SF841	SF841	36 Y	M	The Cader Laboratory
Aged	A1	SF854	SF854	72 Y	M	The Cader Laboratory
	A2	SF180	SF180	60 Y	F	The Cader Laboratory
	A3	SF840	SF840	67 Y	F	The Cader Laboratory
	A4	SF856	SF856	78 Y	F	The Cader Laboratory

The iPSCsNs and iNs were generated at the Neurobiology Lab for Brain Aging and Mental Health (Basel). To ensure neuronal cell consistency, each batch of neuronal cells underwent fluorescence-activated cell sorting (FACS) for positive PSA-NCAM-PE staining or immunostaining for TUJ1 and MAP2 to confirm neuronal marker expression (Supplementary Fig. 7b, c). iPSCs were generated either by TAKARA or Cader’s Lab and were thoroughly characterized according to their highest standards. The quality of neural progenitor cells (NPCs) was verified using the Human Neural Stem Cell Immunocytochemistry Kit (Supplementary Fig. 7a). We followed well-defined, standardized protocols for generating our neuronal models (iPSCsN and iNs), ensuring consistency in neuronal subtype and maturity. Multiple cell batches were used to avoid batch-specific bias, and experiments were normalized to cell number or cellular area to minimize inter-batch variability. We synchronized the cell culture system by seeding comparable cell models at the same density and maintaining consistent culture conditions throughout all experiments. We also strongly viewed the passage of the cell lines to prevent in vitro aging. HFs were only used for mitochondrial and telomeric analyses if they were under passage 10, following established literature guidelines^103,104^. We regularly checked for changes in HF proliferation rates to detect potential culture-induced aging. For the lentiviral transduction to generate iNs, we kept the passage of HFs below P10 to ensure efficient transduction, as higher passages reduced transduction efficacy. iPSCs were maintained at low passages after thawing to preserve culture consistency, though they are generally less sensitive to passage number compared to HFs. Overall, the uniformity of our cellular models was regularly monitored through assessments of cell proliferation, morphology, and overall health by visual inspection. All cells were constantly checked for mycoplasma using the MycoAlert® PLUS Mycoplasma Detection Kit.

#### Primary human fibroblasts (HFs)

HFs were cultured in a growing medium (DMEM with 1% Penicillin-Streptomycin, 1% Glutamax, and 20% tetracycline-free FBS). The cultivation process was carried out at 37 °C and 5% CO_2_ in a humidified incubator. The HFs were grown on 10 cm^2^ dishes and were split at a ratio of 1:3 at a confluency of 80%–100%. The HFs were synchronized 1 day before the experiments were carried out using the serum shock technique^105^. HFs were plated into either FBS precoated 96-well plates at a density of 1.5 × 104 cells per well, FBS precoated XF24 cell culture microplate plates at a density of 2 × 104 cells per well, or 12-well plates with coverslips at a density of 1 ×104 cells per well, depending on the experiments.

#### Directly converted neurons (iNs) from primary human fibroblasts

iNs were obtained from HFs following the approach previously outlined by Zhou-Yang and colleagues, with some minor adjustments^106^. The transduction occurred in TMF medium composed of DMEM with 20% tetracycline-free fetal bovine serum and 0.1% NEAA. The transduced HFs were cultured in TMF containing puromycin (1 mg/ml) for at least five passages. For the neuronal conversion, 100% confluent UNA-transduced HFs were pooled into a 3:1 density on 6-well plates. After 1 day, the medium was exchanged to neuronal conversion medium (NK medium) containing DMEM/F-12, Neurobasal-A, 1xB-27, 1xN-2, 1 μg/ml Laminin, 400 μg/ml db-cAMP, 2 μg/ml doxycycline, 150 ng/ml Noggin, 0.5 μM LDN-193189, 0.5 μM A83-1, 3 μM CHIR-99021, 5 μM Forskolin, 10 μM SB-431542, 1 μM Pyrintegrin, 7.5 μM KC7F2, 0.1 μM AZ960, and 0.75 μM ZM 33637. The NK medium was replaced every other day for 3–4 weeks. Following the conversion, the cells were dissociated with TrypLE™ Select Enzyme and sorted by FACS. Sorting of iNs involved positive PSA-NCAM-PE (neuronal staining) and DAPI labeling (dead cells) in a buffer with 150 mM myo-inositol, 5 mg/ml polyvinyl alcohol, 1% DNase, and 10 μM Rock-inhibitor in PBS using the FACS Sorter Aria III (FACS Core Facility, Biozentrum, University of Basel). The sorted iNs were gathered in NK medium containing Rock-inhibitor (Y-27632) and z-VAD-FMK and plated either on Geltrex-precoated μ-Slide 8 well ibidi chambers, Seahorse XFp Cell Culture Miniplate at a density of 2.5 × 10^4^ cell/well or on 96-well plates at a density of 5 × 10^4^ cell/well, depending on the experiments. The next day, the medium was replaced with neuronal maturation media consisting of BrainPhys, 1xB-27, 1xN-2, 1 μg/ml Laminin, 500 μg/ml db-cAMP, 20 ng/ml GDNF, and 20 ng/ml BDNF and refreshed every 24 h. Following 72 h of plating, measurements were performed.

#### Induced pluripotent stem cells (iPSCs)

iPSCs were cultured in feeder-free conditions and on Cellartis DEF-CS COAT-1-coated plates using the Cellartis DEF-CS culture system, following the manufacturer’s instructions (Takara Bio)^107^.

#### Induced pluripotent stem cell-derived neurons (iPSCsNs)

The PSC Neural Induction protocol by Gibco was conducted to convert iPSCs in NPCs. At the 6-h mark (day 0), the Cellartis DEF-CS culture system medium was replaced with neural induction medium (NIM, Neurobasal Medium minus phenol red and 2% Neural Induction Supplement). On day seven, primitive NPCs were dissociated using StemPro Accutase and placed onto Geltrex-coated 6-well cell culture plates (4.8–9.6 × 10^5^ cells per well) in neural expansion medium (NEM; 1:1 ratio of Neurobasal Medium to Advanced DMEM/F-12, 2% Neural Induction Supplement, and 10 μM Y-27632). The expanded NPCs were either cryopreserved in NEM with 10% DMSO or converted into iPSCsNs using Takara Bio’s protocol. NPCs were cultivated on Geltex-coated 6-well plates using RHB-A media with 20 ng/ml EGF and 20 ng/ml FGF for at least two passages. StemPro Accutase with 10 μM ROCK Inhibitor was used for passaging NPCs and placed in Geltrex-coated 10 cm^2^ cell culture dishes with a density of 4.5 × 10^5^ cells per dish. The cells were cultured in RHB-BASAL medium with 0.5% NDiff N2, 1% B-27 Supplement, and 10 ng/ml FGF for 6 days. On the 7th day, the differentiation medium was changed to a mixture of RHB-BASAL medium and Neurobasal Medium without phenol red in a 1:1 ratio with 0.25% 1xN-2, 1% B-27 Supplement, 10 ng/ml FGF, and 0.5% GlutaMAX. On day 14, the differentiation medium was changed to Neurobasal Medium without phenol red, 2% B-27 Supplement, 1% GlutaMAX, and 20 ng/ml BDNF for 14 days. The iPSCsNs were transferred to the appropriate assay plates, either Geltrex-coated 96-well cell culture plates (6.0 × 10^5^ cells per well), Geltrex-coated 96-well plates (6.0 × 10^5^ cells per well), or Geltrex-coated Seahorse XFp Cell Culture Miniplate (3.0 × 105 cells per well). The iPSCsNs were cultured in Neurobasal Medium without phenol red, supplemented with 2% B-27 Supplement, 1% GlutaMAX, and 20 ng/ml BDNF with 10 μM of the ROCK Inhibitor (O/N) for 7 days.

### Cellular ATP level

For the determination of the total ATP content in the cells, the bioluminescence assay ATPlite 1step was conducted following the manufacturer’s instruction^108^. The cells were plated on precoated transparent 96-well cell culture plates at the corresponding density. The emitted light was measured using the Cytation 3 Cell Imaging Multi-Mode Reader.

### Mitochondrial membrane potential (MMP)

The fluorescent dye TMRM was used to measure the MMP^107^. The cells were seeded at the appropriate densities onto black 96-well cell culture plates and incubated with 0.4 μM TMRM for 30 min at 37 °C and 5% CO_2_. The fluorescence at 548 nm (excitation) and 574 nm (emission) was then measured using the Cytation 3 Cell Imaging Multi-Mode Reader.

### Superoxide anion radical levels and mitochondrial reactive oxygen species (ROS)

DHR was used to measure the mitochondrial ROS level, while MitoSOX was used to detect the mitochondrial superoxide anion radical level (°O_2_^−^)^105^. The cells were carefully plated onto black 96-well cell culture plates and then incubated with 10 μM of DHR for 30 min or 5 μM of MitoSOX for 2 h at 37 °C and 5% CO_2_ in the incubator. Following incubation, the cells were rinsed twice with HBSS. The Cytation 3 Cell Imaging Multi-Mode Reader was then used to measure DHR at 485 nm (excitation)/535 nm (emission) and MitoSOX at 531 nm (excitation)/595 nm (emission).

### NAD^+^ to NADH

Intracellular NAD^+^/NADH levels were measured using an enzyme cycling assay^105,109,110^. Cells were lysed in 250 μl of protein lysis buffer (150 mM Tris, 150 mM NaCl, 1% NP-40, 0.1% SDS, and 2 mM EDTA). NAD+ and NADH were extracted by heat-incubating for 5 min at 95 °C 100 μl of lysate with either 1 M HCl (for NAD+) or 1 M NaOH (for NADH), followed by neutralization and centrifugation at 10,000 × *g* for 10 min at 4 °C to collect the supernatant. The protein content was determined from the remaining lysate. For the cycling assay, the samples were plated on a transparent 96-well plate and incubated with 50 μl of a mixture containing: 0.02 M Tricine-NaOH buffer (pH 8), 8 mM EDTA, 0.84 mM MTT, 3.32 mM PES, and 1 M Ethanol for 10 min at 37 °C. After this step, alcohol dehydrogenase (10 U/ml) was added and incubated at 37 °C for 1 h. The reaction was stopped with 50 μl NaCl (6 M). In the last step, 100 μl ethanol (96%) was added to solubilize the formazan. The absorbance was detected at 595 nm using the Cytation 3 Cell Imaging Multi-Mode Reader.

#### Determination of protein content—Lowry assay

The protein content was determined using the DCTM Protein Assay Kit, following the manufacturing script^107^. The absorbance was then read at 690 nm using the Cytation 3 Cell Imaging Multi-Mode Reader.

### Mitochondrial respiration and glycolysis

The Seahorse analyzer allows real-time quantification of OCR and ECAR. The sequential injection of mitochondrial modulators enabled the examination of mitochondrial respiration parameters: basal respiration, ATP turnover, maximal respiration, and respiratory reserve capacity (Table 3)^105,111^. The sequential administration of glycolytic modulators enabled the identification of glycolysis parameters, including glycolysis itself, glycolytic capacity, and non-glycolytic acidification (Table 4).

**Table 3:** Mitochondrial stress test parameters

Assay parameter	Equation	Definition
Non-Mitochondrial Oxygen Consumption	Minimum rate after rotenone/antimycin A injection	OCR from non-mitochondrial sources; essential for accurate mitochondrial measurements.
Basal Respiration	(Mean measurements before first injection)−(Non-Mitochondrial Oxygen Consumption)	Baseline oxygen consumption for ATP production via mitochondria.
ATP-Production Coupled Respiration	(Basal Respiration)−(Proton Leak)	Portion of basal respiration used for ATP synthesis; indicated by OCR drop after oligomycin.
Maximal Respiration	(Maximum rate after FCCP injection)−(Non-Mitochondrial Oxygen Consumption)	Highest achievable OCR after FCCP uncoupling.
Spare Respiratory Capacity	(Maximal Respiration)−(Basal Respiration)	Difference between maximal and basal respiration; indicates the cell’s ability to meet increased energy demands.

**Table 4:** Glycolysis stress test parameters

Assay parameter	Equation	Definition
Non-Glycolytic Acidification	Minimum rate measurement after 2-DG injection	ECAR from non-glycolytic sources; essential for accurate glycolysis detection.
Basal glycolysis or glycolysis	(Maximum rate measurement between glucose and oligomycin injection)−(Non-Glycolytic Acidification)	Baseline glycolysis rate after glucose addition.
Glycolytic Capacity	(Maximum rate measurement between Oligomycin and 2-DG injection)−(Non-Glycolytic Acidification)	Maximum glycolysis rate when OXPHOS is inhibited, forcing cells to rely entirely on glycolysis.

The Seahorse XFe24 Analyzer was used to measure the HFs, whilst the Seahorse XF HS Mini Analyzer was employed for both neuronal models. Before measurements, the medium was replaced with Seahorse XF DMEM medium (pH 7.4). For OCR detection, the medium was supplemented with 25 mM glucose, 4 mM glutamine, and 1 mM pyruvate for HFs and iNs, whereby for iPSCsNs, a concentration of 18 mM glucose, 4 mM glutamine, and 2 mM glutamine was added. ECAR was measured without glucose. Seahorse plates were equilibrated in a CO_2_-free incubator at 37 °C for 45–60 min. OCR rates were recorded under basal conditions followed by sequential injection of 1 μM (HFs), 2.5 μM (iNs), or 1.5 μM (iPSCsNs) oligomycin, 2 μM (HFs and iNs) or 1 μM (iPSCsNs) FCCP, and a combination of 4 μM (HFs), 2 μM (iNs), or 0.5 μM antimycin A and 2 μM (HFs and iNs) or 1 μM (iPSCsNs) rotenone. The Seahorse XF Glycolysis Stress Test was conducted to identify glycolysis by detecting the ECAR, following the directions provided by the manufacturer. The ECAR was measured under basal conditions, followed by 25 mM glucose (HFs and iNs) or 18 mM (iPSCsNs), 1 μM (HFs), 2.5 μM (iNs), or 1.5 μM (iPSCsNs) oligomycin, and 25 mM (HFs and iNs) or 50 mM 2-deoxy-glucose by sequential injection.

### Mitochondrial network morphology

**Fig. 8:**
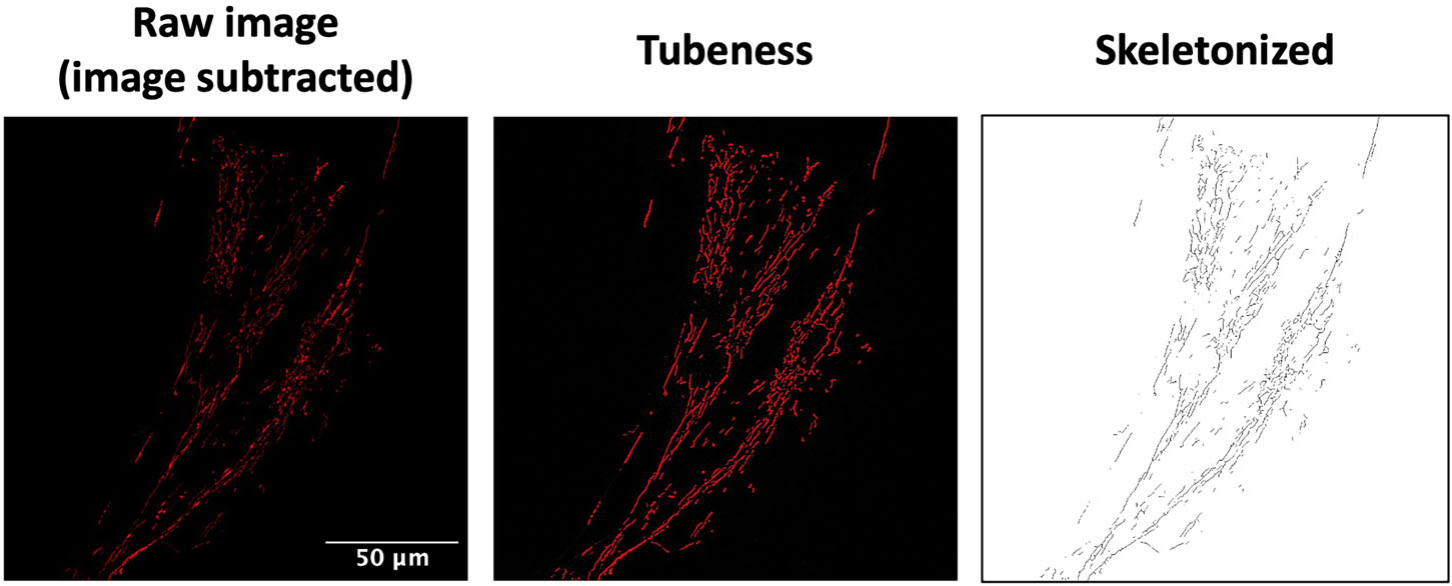
Representative z-projection microscopy images of the mitochondrial network stained with the TOMM20 antibody in young HFs (×63 magnification). The left panel shows the raw image after background subtraction (rolling ball = 5 pixels). The middle panel (“tubeness”) displays the same images processed with the morphometry macro in FIJI software, as mentioned in ref. ^112^. The right panel (“skeletonized”) presents the binary representations of the mitochondrial network (in black) after further image processing using FIJI’s “Skeletonize” function.

To observe the structure of the mitochondrial network morphology, HFs were plated onto FBS-precoated coverslips, and both neuronal models onto Geltrex-precoated μ-Slide 8 well ibidi chambers or 96-well plates. The assessment was conducted as done in Szabo et al.^107^. The cells were fixed with 4% formaldehyde for 15 min and then rinsed twice with PBS^+ Mg, + Ca^. To permeabilize the cells, 0.2% Triton X-100 for 15 min was used and then blocked with 2% BSA for 1 h. The mitochondria were stained with the mitochondrial marker translocase of the outer mitochondrial membrane complex subunit 20, TOMM20 Alexa Fluor® 555, for 4 h at room temperature. The coverslip with the HFs was mounted using the Vectashield H-1000 mounting medium, and the neurons were visualized in PBS. The microscopy images for HFs and iNs were obtained using an inverted microscope (Leica Microsystems TCS SPE DMI4000) connected to an external light source (Leica EL6000). The images were taken using an x63 oil immersion objective. Before examining the mitochondrial structure in the iNs, the images were subjected to deconvolution using Huygens Deconvolution Software to minimize nonspecific signals. The iPSCsNs were visualized by the Nikon Inverted Research Microscope Eclipse Ti2-E using the ×60 oil immersion objective. Further, the iPSCsNs were stained with NucBlue™ Fixed Cell ReadyProbes™ Reagent (DAPI) to visualize the nucleus, and the Nikon NIS-HC software was used. An acquisition of an XYZ-confocal image with maximum intensity projection was utilized for all cell types. The acquisition settings within each cell type were consistent throughout the imaging process. The morphological investigation included blind examination using the NIH ImageJ program^112^, which was applied to the whole image (Fig. 8). The changes in the analysis settings were set for the rolling ball as 5 for iNs and HFs, whereas for iPSCsNs, a rolling ball of 10 was chosen. All data were obtained within the dynamic range of each camera, and all measurements were conducted on the unprocessed photos. The figures in the article have been modified to enhance clarity, ensuring that all representative pictures are processed similarly. Representative images of the mitochondrial network morphology of each donor are shown in Supplementary Fig 8.

### Mitochondrial mass

To quantify the mitochondrial mass, the mitochondria were stained using the mitochondrial dye MitoTracker™ Green FM. The CellTracker™ Blue CMAC Dye was used to standardize the fluorescence signal to identify the cellular surface area. All cell models were stained with MitoTracker™ Green FM (0.1 μM) and CellTracker™ Blue CMAC Dye (5 μM) for 1 h at 37 °C and 5% CO_2_. Subsequently, the cells underwent two washes with HBSS. The fluorescence signals of MitoTracker™ Green FM at 490 nm (excitation) and 516 nm (emission), as well as CellTracker™ Blue CMAC Dye at 353 nm (excitation) and 466 nm (emission), were measured at the same time using the Cytation 3 Cell Imaging Multi-Mode Reader. The mitochondrial mass was determined by calculating the mitochondria-to-cellular mass ratio.

### Telomere length

#### DNA isolation

DNA was isolated using the FlexiGene® DNA KIT (250) according to the FlexiGene DNA Handbook. DNA content was assessed using the Take3TM Microvolume Plate in combination with the Cytation 3 Cell Imaging Multi-Mode Reader, and the DNA samples were stored at −20 °C until further use.

#### Telomere length determination by qPCR

The telomere length was measured by quantitative polymerase chain reaction (qPCR)^113,114^. Table 5 lists the forward and reverse primers of TELO and SCG (ß-globin). The composition of one reaction of TELO master mix was 1.08 μl teloF primer (54 nM), 1.6 μl teloR primer (80 nM), 5.32 μl H_2_O, 10 μl of GoTaq® qPCR Master Mix, and 0.2 μl Supplemental CXR Reference Dye and for SCG master mix was 1.6 μl ß-globinF primer (80 nM), 1.6 μl ß-globinR primer (80 nM), 4.8 μl H_2_O 10 μl of GoTaq® qPCR Master Mix, and 0.2 μl Supplemental CXR Reference Dye. DNA samples were added to the 96-well PCR plates at 10 ng/μl concentration. The qPCR was run with an initial denaturation at 95 °C for 10 min, followed by 50 cycles of 95 °C for 10 s and 58 °C for 60 s. The Ct values were analyzed using the comparative Ct method (2^−∆∆Ct^) and represented by the T/S ratio, with a mixture of HFs DNA samples as the internal control.

**Table 5:** Forward and reverse primer sequences of TELO and SCG

Primer	Sequence (5′–3′)
teloF	CGGTTTGTTTGGGTTTGGGTTTGGGTTTGGGTTTGGGTT
teloR	GGCTTGCCTTACCCTTACCCTTACCCTTACCCTTACCCT
ß-globinF	GCTTCTGACACAACTGTGTTCACTAGC
ß-globinR	CACCAACTTCATCCACGTTCACC

### Telomerase activity

Telomerase Repeat Amplification Protocol (TRAP) was performed to determine the telomerase activity^115–117^. A defined amount of cells (HFs and iPSCs: 10,000 cells; iNs and iPSCsN: 100,000 cells) were incubated in ice-cold NP-40 lysis buffer (10 mM Tris-HCl (pH 8.0); 1 mM MgCl_2_(6 H_2_O); 1 mM EDTA, 1% (v/v) NP-40, 0.25 mM sodium deoxycholate, 10% (v/v) glycerol, 150 mM NaCl, 5 mM 2-mercaptoethanol, and 0.1 mM AEBSF) for 30 min on ice. The samples were then centrifuged for 20 min at 14,000 rpm at 4 °C. The supernatant was collected and further utilized in the TRAP assay. A master mix containing 0.2 μl ACX- primer (300 nM), 0.2 μl TS Primer (300 nM), 7.6 μl DNase/RNase-free water 10 μl of GoTaq® qPCR Master Mix, and 0.2 μl Supplemental CXR Reference Dye per one reaction was prepared. The primer sequences are listed in Table 6. The master mix (18 μl per well) was loaded on a 96-well PCR plate. Followed by 2 μl of the lysate was added to the corresponding well. A positive control of HEK293 cells in the range of 10,000 cells to 640,000 cells was measured on every PCR plate. The preparation of the TRAP assay was done on ice. The real-time PCR was performed using the Step One Plus system. The settings of the measurement were 25 °C for 40 min, 95 °C for 10 min, followed by cycling of 40 repeats of (1) 30-s hold at 95 °C, (2) 30-s hold at 52 °C, and (3) 45-s at 72 °C. The Ct values were exported using the SABiosciences PCR Array Data Analysis Software. The final data were represented as the normalization to the positive control.

**Table 6:** Primer sequences of the TRAP assay

Primer	Sequence (5′–3′)
TS primer	AATCCGTCGAGCAGAGTT
ACX primer	GCGCGGCTTACCCTTACCCTTACCCTAACC

### Gene expression

#### RNA isolation

The RNA was isolated using the RNeasy Mini Kit protocol. RNA content was assessed via a Nanodrop 1000 spectrophotometer. Then, RNA samples were either directly converted to cDNA or stored at −80 °C.

#### Reverse transcription and gene expression

The extracted RNA was transformed to cDNA using the RT^2^ First Strand Kit^118^ by following the manufacturer's instructions to measure the gene expression in the neurons. Next, the gene expression was detected using the Custom RT^2^ Profiler PCR Arrays with the RT^2^ SYBR Green ROX qPCR Master Mix according to the manufacturer’s guidelines. The RT^2^ Profiler PCR Arrays were precoated with custom-chosen primers (primers listed in Table 7). The Step One Plus system was used to perform the quantitative real-time PCR. The CT values (automatically generated by the Step One Plus software) were exported and analyzed with the GeneGlobe Data Analysis Centre Software and MetaboAnalyst 5.0.

**Table 7:** List of genes assessed with the Custom RT^2^ Profiler PCR Arrays (Qiagen)

Gene symbol	Assay Nr.	GeneBank
PPARGC1A	PPH00461F	NM_013261
TFAM	PPH09934B	NM_003201
SIRT3	PPH22989A	NM_012239
PARP1	PPH00686B	NM_001618
PFKFB3	PPH00024F	NM_004566
TP53	PPH00213F	NM_000546
FIS1	PPH19947A	NM_016068
OPA1	PPH12084A	NM_130837
MFN2	PPH18422A	NM_014874
MFN1	PPH20215A	NM_033540
DNM1L	PPH13763A	NM_005690
BCL2	PPH00079B	NM_000633
SOD1	PPH00234B	NM_000454
CAT	PPH00420B	NM_001752
GPX1	PPH00154F	NM_000581
MTOR	PPH02311D	NM_004958
AKT1	PPH00088B	NM_005163
SIRT1	PPH02188A	NM_012238
UCP2	PPH00476F	NM_003355
NFE2L2	PPH06070A	NM_006164
PRKAA2	PPH15207A	NM_006252
LDHA	PPH02047H	NM_005566
SQSTM1	PPH02107A	NM_003900
FOXO1	PPH01964F	NM_002015
GAPDH	PPH00150F	NM_002046

### Normalization

After each experiment, the cells were fixed with 4% formaldehyde for 10 min and washed twice with PBS. Followed by staining with DAPI for 5 min; the cells were washed once with PBS. The cell number was determined using the cell analyzing settings on the Gen5 Image 3.11 software of the Citation 3 Cell Imaging Multi-Mode Reader. The average cell count from a parallel running plate was utilized for the cellular ATP level. The NAD^+^ and NADH values were normalized to the protein level. The mitochondrial mass was standardized to the cell surface.

### Statistics and reproducibility

Data management was performed using GraphPad Prism 10 (version 10.2.3), Excel software, R-software, and the metaboanalyst.ca website. PCA was conducted to visually represent the primary directions that most accurately characterize the variability in our dataset without explicitly referring to group labels. A heat map was generated from a hierarchical cluster analysis using Euclidean distance and Ward’s linkage method (Supplementary Fig. 4). To generate the radar charts for the donor comparison, the statistical software R (version 4.2.1), including the packages fmsb (version 0.7.5)^119^, was used. Each experiment was performed in three to five independent biological replicates per donor and condition, with technical replicates included within each experiment. For statistical analysis, a non-parametric Mann–Whitney *U* test, a two-way ANOVA was used to compare multiple parameters, or an unpaired Student’s *t*-test was applied. Detailed statistical information, including exact *n* values and test types, is provided in the respective figure legends. Statistical parameters, including the number of values, minimum, maximum, range, mean, standard deviation, and standard error of the mean for each donor across all experiments, are presented in Supplementary Data 2–6. Comparisons of individual data points between young and aged HFs, iNs, and iPSCsNs are presented in Supplementary Figs. S1–S3.

### Reporting summary

Further information on research design is available in the Nature Portfolio Reporting Summary linked to this article.

## Supplementary information

Supplementary Material

Description of Additional Supplementary Files

Supplementary Data 1–6

Reporting Summary

## Data Availability

All data supporting the findings of this study are available through the Open Science Framework (OSF) at the following link: https://osf.io/xs6cr/.
