# Supplementary material for: Tracing mitochondrial marks of neuronal aging in iPSCs-derived neurons and directly converted neurons
Source: Commun Biol. 2025 May 10;8:723. doi: 10.1038/s42003-025-08152-2 (PMC12064796; doi:10.1038/s42003-025-08152-2)
Supplement: Supplementary file 1 — Supplementary Material [file 42003_2025_8152_MOESM1_ESM.pdf]

Tracing Mitochondrial Marks of Neuronal Aging in iPSCs-Derived Neurons and Directly Converted Neurons

Nimmy Varghese[1,2,†], Leonora Szabo[1,2], M. Zameel Cader[3], Imane Lejri[1,2], Amandine Grimm[1,2,4], Anne Eckert[1,2,*]

[1]Research Cluster Molecular and Cognitive Neurosciences, University of Basel, 4002 Basel, Switzerland

[2]Neurobiology Lab for Brain Aging and Mental Health, University Psychiatric Clinics Basel, 4002 Basel, Switzerland

[3]Nuffield Department of Clinical Neuroscience, University of Oxford, Oxford, UK

[4]Department of Biomedicine, University of Basel, 4055 Basel, Switzerland

[†]Present address: Max Planck Florida Institute for Neuroscience, Jupiter, FL, 33458, USA

[*] Corresponding author. Tel: +41(0) 61 325 54 87; E-mail: anne.eckert@unibas.ch

Supplementary Information

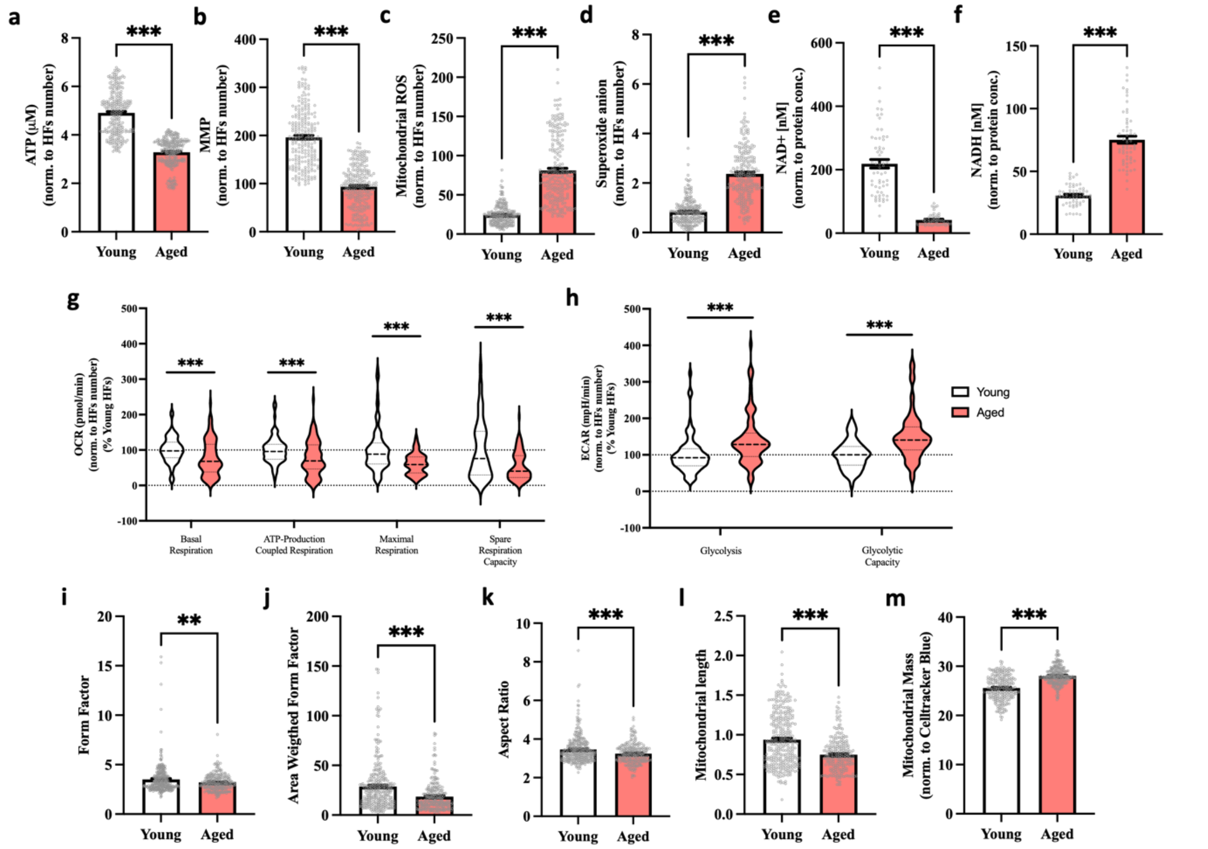

Figure S1: Comparisons of individual data points between young and aged HFs

a, Cellular ATP level (N= 5 independent experiments, n=13-14 replicates per experiment). **b**, MMP level (N= 5 independent experiments, n=10-17 replicates per experiment). **c**, Mitochondrial ROS (N= 5 independent experiments, n=11-12 replicates per experiment). **d**, Mitochondrial superoxide anion (N= 5 independent experiments, n=11-12 replicates per experiment). **e,f**, Cellular NAD+ content (**e**) and NADH content (**f**) (N= 5 independent experiments, n=3 replicates per experiment). **g**, Basal respiration, ATP-production coupled respiration, maximal respiration, and spare respiration capacity. (N= 5-6 independent experiments, n=3-5 replicates per experiment). **h**, Glycolysis and glycolytic capacity. (N= 4-6 independent experiments, n=2-4 replicates per experiment). **i-l**, Form Factor (**i**), Area Weighted Form Factor (**j**), Aspect Ratio (**k**), and Length (**l**). (N= 4-5 independent experiments, n=12-33 replicates per experiment). **m,** Mitochondrial mass (N= 4 independent experiments, n=15-16 replicates per experiment). Data information: All data are represented as the mean ± SEM of each 4 different young and aged HFs. Statistical parameters, including the number of values, minimum, maximum, range, mean, standard deviation, and standard error of the mean are presented in Supplementary Table 2. Values were normalized on the cell count. Student's unpaired t-test was performed for young HFs versus aged HFs (* $p < 0.05$, ** $p < 0.01$, *** $p < 0.001$).

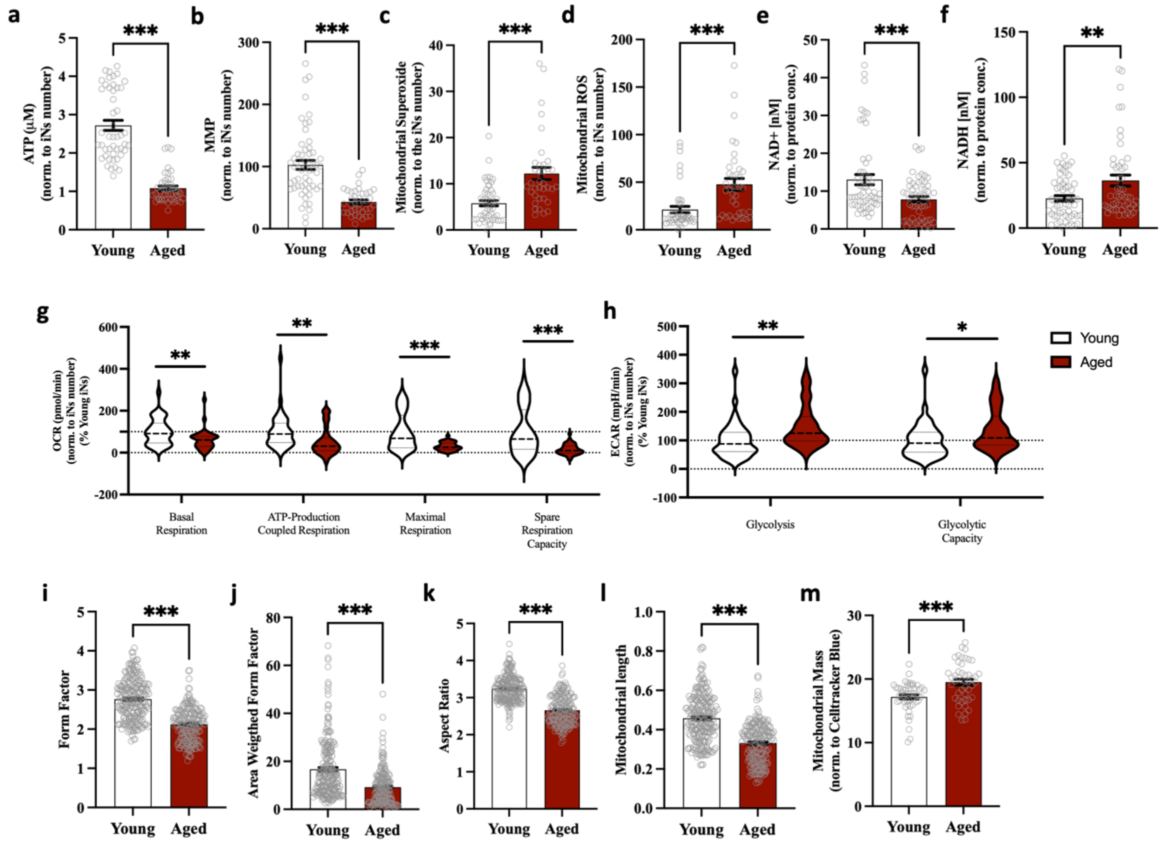

Figure S2: Individual data point comparison between young and aged iNs

a, Cellular ATP level (N= 3-4 independent experiments, n=2-3 replicates per experiment). **b**, MMP (N= 4-5 independent experiments, n=2-3 replicates per experiment). **c**, Mitochondrial superoxide anion (N= 4-5 independent experiments, n=2-3 replicates per experiment). **d**, Mitochondrial ROS (N= 4-5 independent experiments, n=2-3 replicates per experiment). **e**, **f**, Cellular NAD^+ content (**e**) and NADH (**f**) content (N= 4-5 independent experiments, n=2-3 replicates per experiment). **g**, Basal respiration, ATP-production coupled respiration, proton leak, maximal respiration, and spare respiration capacity. (N= 4-5 independent experiments, n=2-3 replicates per experiment). **h**, Glycolysis, glycolytic capacity, and glycolytic reverse. (N= 3-5 independent experiments, n=2-3 replicates per experiment). **i-l**, Calculated mitochondrial parameters, Form Factor (**i**), Area Weighted Form Factor (**j**), Aspect Ratio (**k**), and Length (**l**). (N= 4-5 independent experiments, n=12-33 replicates per experiment). **m,** Mitochondrial Mass (N= 4-5 independent experiments, n=2-3 replicates per experiment). Data information: All data are represented as the mean ± SEM of each of the four different young and aged iNs donors. Statistical parameters, including the number of values, minimum, maximum, range, mean, standard deviation, and standard error of the mean are presented in Supplementary Table 2. Only three donors were assessed for gene expression using three technical replicates. Values were normalized on the cell count. The representative images were chosen for visualization purposes. Student's unpaired t-test was performed for young iNs versus aged iNs (* $p < 0.05$, ** $p < 0.01$, *** $p < 0.001$).

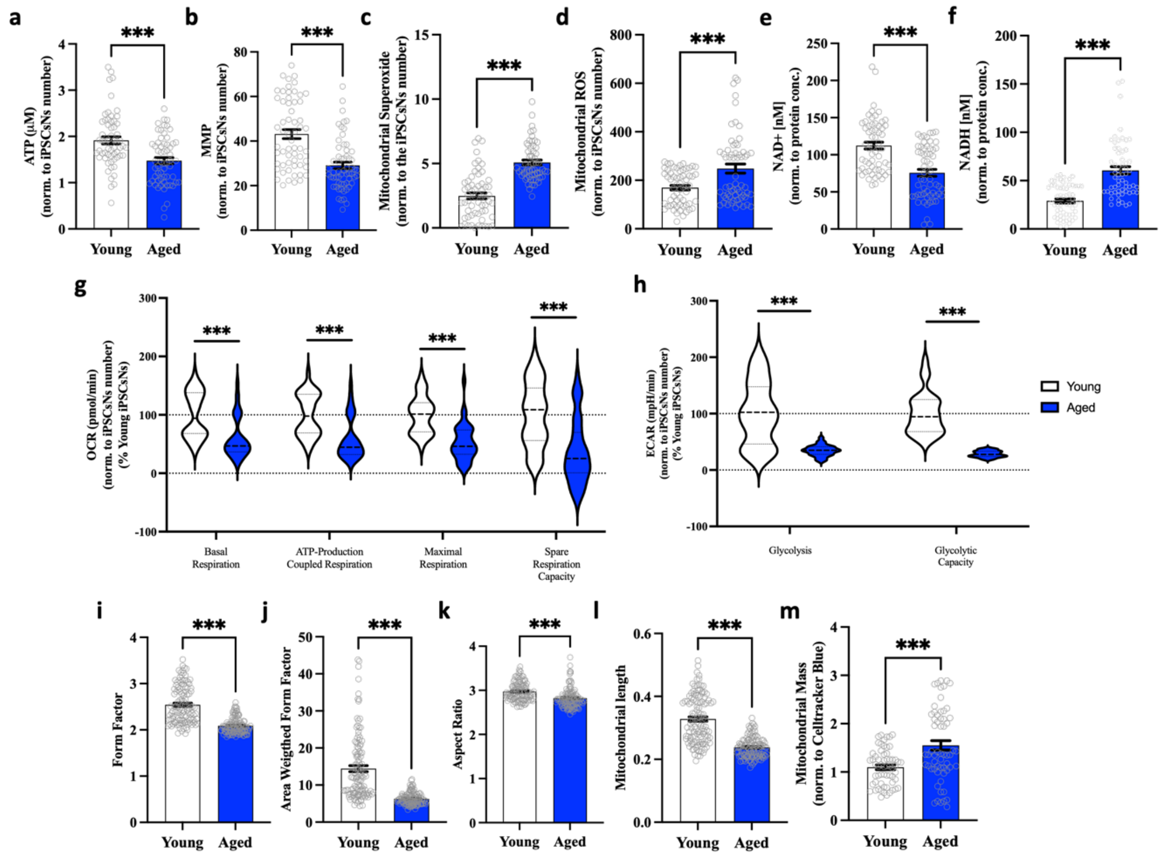

Figure S3: **Comparisons of individual data points between young and aged iPSCsNs**

a, Cellular ATP level (N= 5 independent experiments, n=2-3 replicates per experiment). **b,** MMP level (N= 5 independent experiments, n=2-3 replicates per experiment). **c,** Mitochondrial superoxide anion (N= 5 independent experiments, n=3 replicates per experiment). **d,** Mitochondrial ROS (N= 5 independent experiments, n=3 replicates per experiment). **e,f,** Cellular NAD^{+} content (**e**) and NADH content (**f**) (N= 5 independent experiments, n=3 replicates per experiment). **g,** Basal respiration, ATP-production coupled respiration, maximal respiration, and spare respiration capacity. (N= 4 independent experiments, n=2-3 replicates per experiment). **h,** Glycolysis and glycolytic capacity. (N= 3 independent experiments, n=2-3 replicates per experiment). **i-l**, Calculated mitochondrial parameters, Form Factor (**i**), Area Weighted Form Factor (**j**), Aspect Ratio (**k**), and Length (**l**). (N= 5 independent experiments, n=3 replicates per experiment). **m,** Mitochondrial Mass (N= 5 independent experiments, n=3 replicates per experiment). Data information: All data are represented as the mean ± SEM of each of the four young and aged iNs. Statistical parameters, including the number of values, minimum, maximum, range, mean, standard deviation, and standard error of the mean are presented in Supplementary Table 2. Only three donors were assessed for gene expression with three technical replicates. Values were normalized on the cell count. The representative images were produced for visualization purposes. Student's unpaired t-test was performed for young iNs versus aged iNs (* $p < 0.05$, ** $p < 0.01$, *** $p < 0.001$).

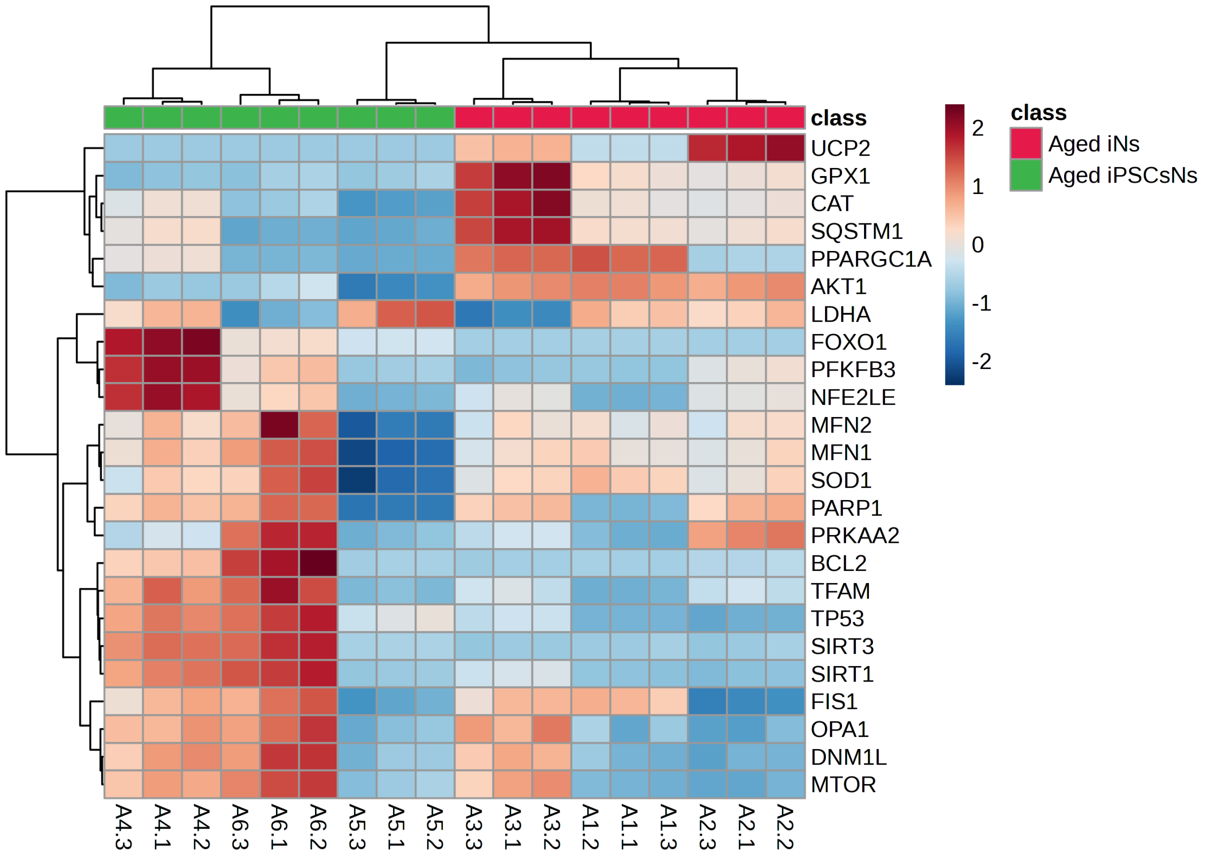

Figure S4: Investigation of differences in aged iNs versus aged iPSCsNs using quantitative real-time PCR analysis. Clustering results are shown as a heatmap of aged iNs vs. aged iPSCsN (distance measured using Euclidean, and clustering algorithm using ward.D). **Data information**: The figure was generated on metaboanalyst.ca. The represented values were normalized by autoscaling (mean-centered and divided by the standard deviation of each variable). The data were represented as Gene expression ($2^{(-Avg.(Delta(Ct))}$) by using the Houskeeping gene GAPDH.

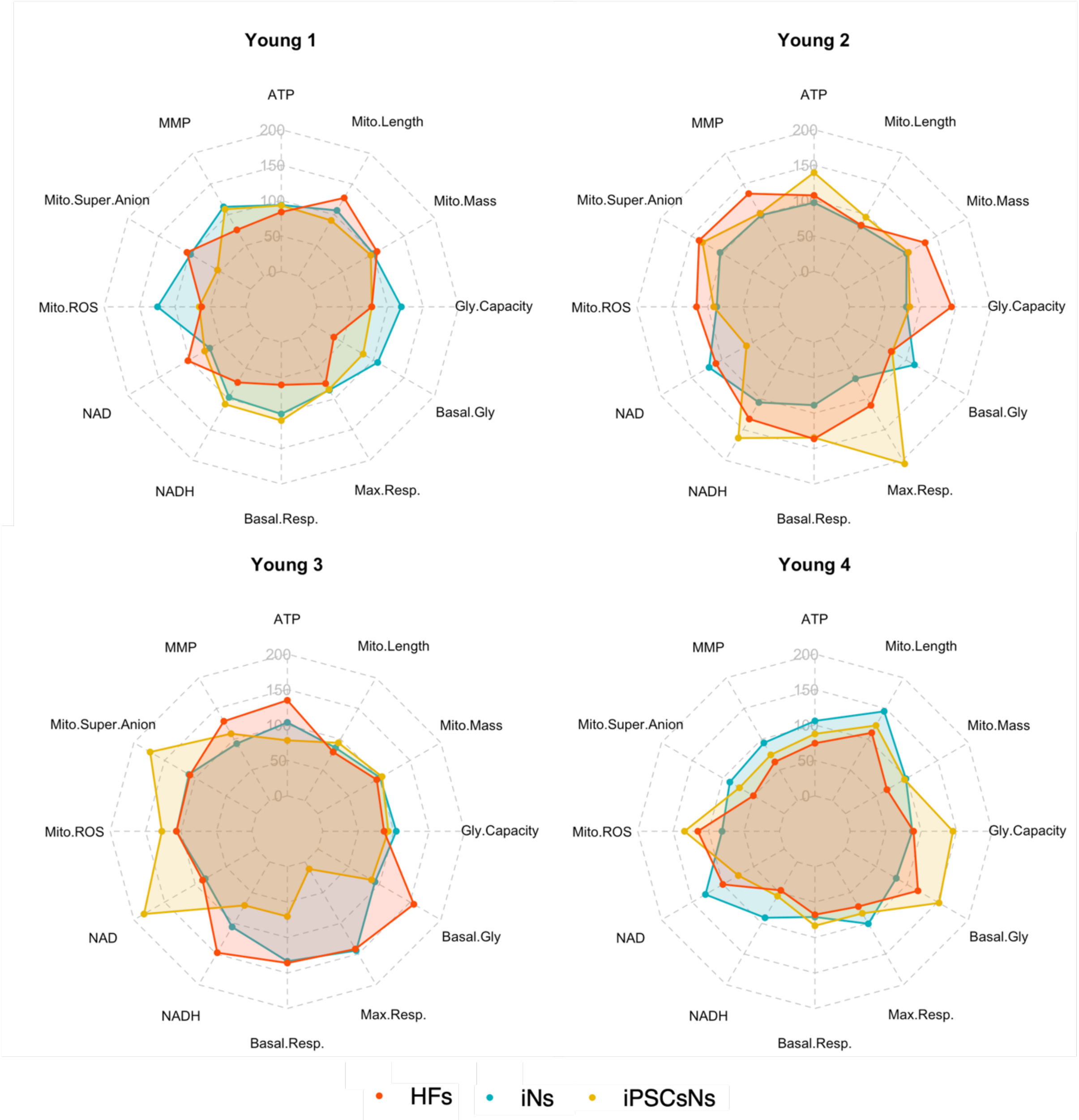

Figure S5:**The radar plot summarizes values from each young donor and shows the different cell models of the same donor in a single graph.** The radar plot was generated with the R-software. The data used to generate the radar plot are represented as % a percentage of the mean young.

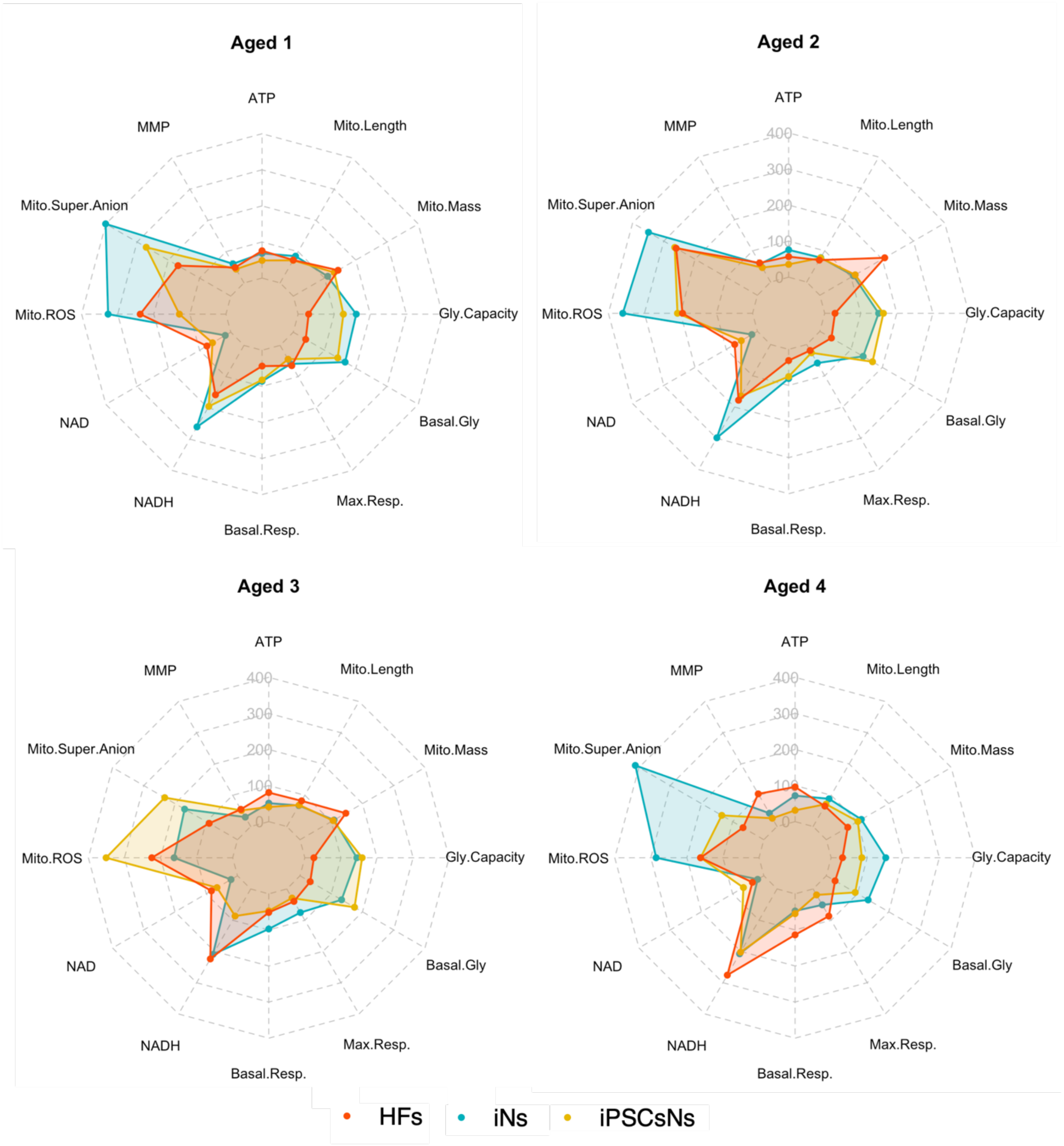

Figure S6: The radar plot summarizes values from each aged donor and represents the different cell models in the same graph. The radar plot or spider web were generated with the R-software. The data used to generate the radar plot are the represented as % percentage to the mean young.

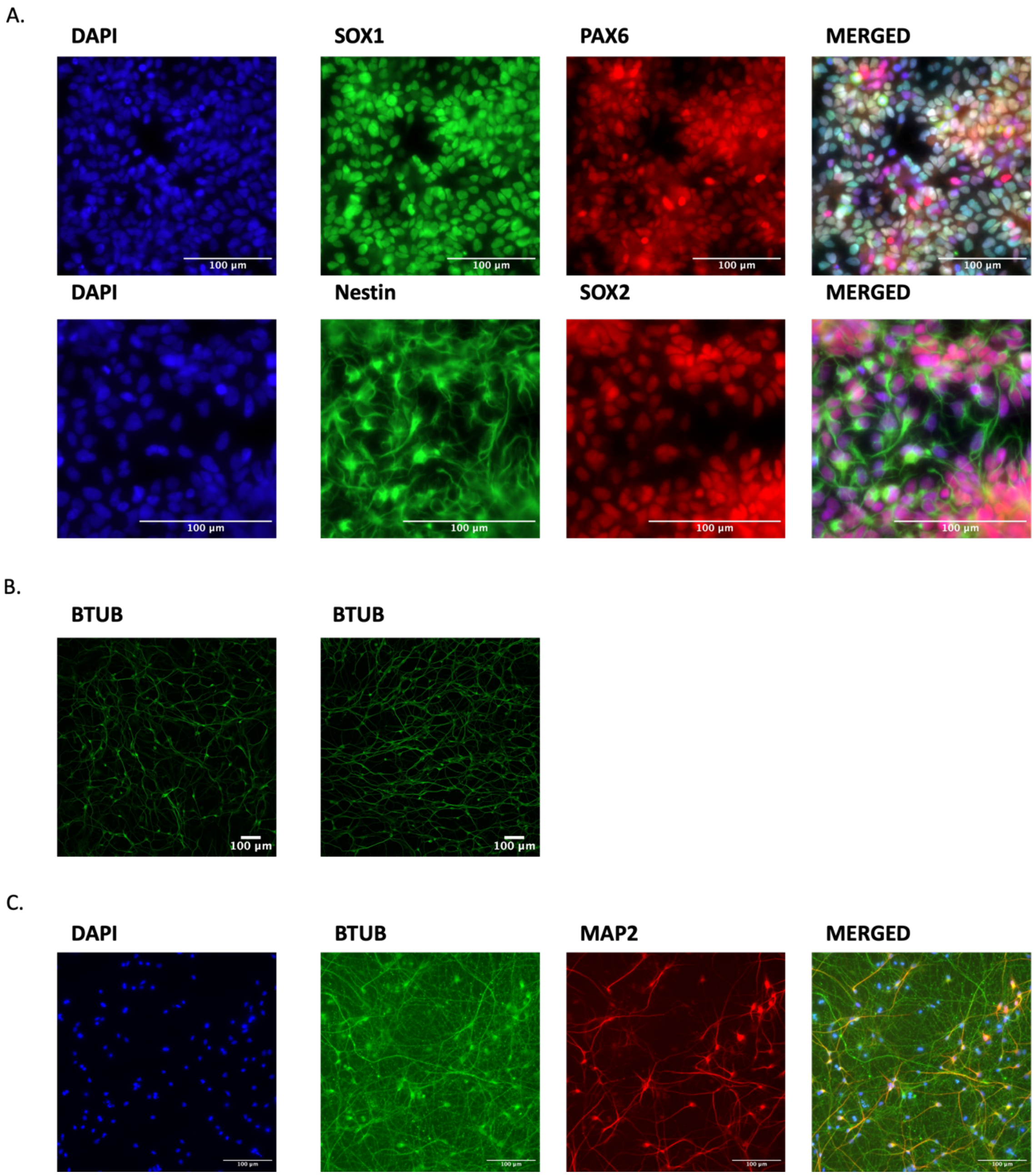

Figure S7: Immunostaining a, Quality assessment of NPCs using the Neural Stem Cell Immunocytochemistry Kit, with staining for SOX1, SOX2, Nestin, and PAX6, **b**, BTUB staining in iNs, and **c**, dual staining for BTUB and MAP2 in iPSCsNs to confirm neuronal identity.

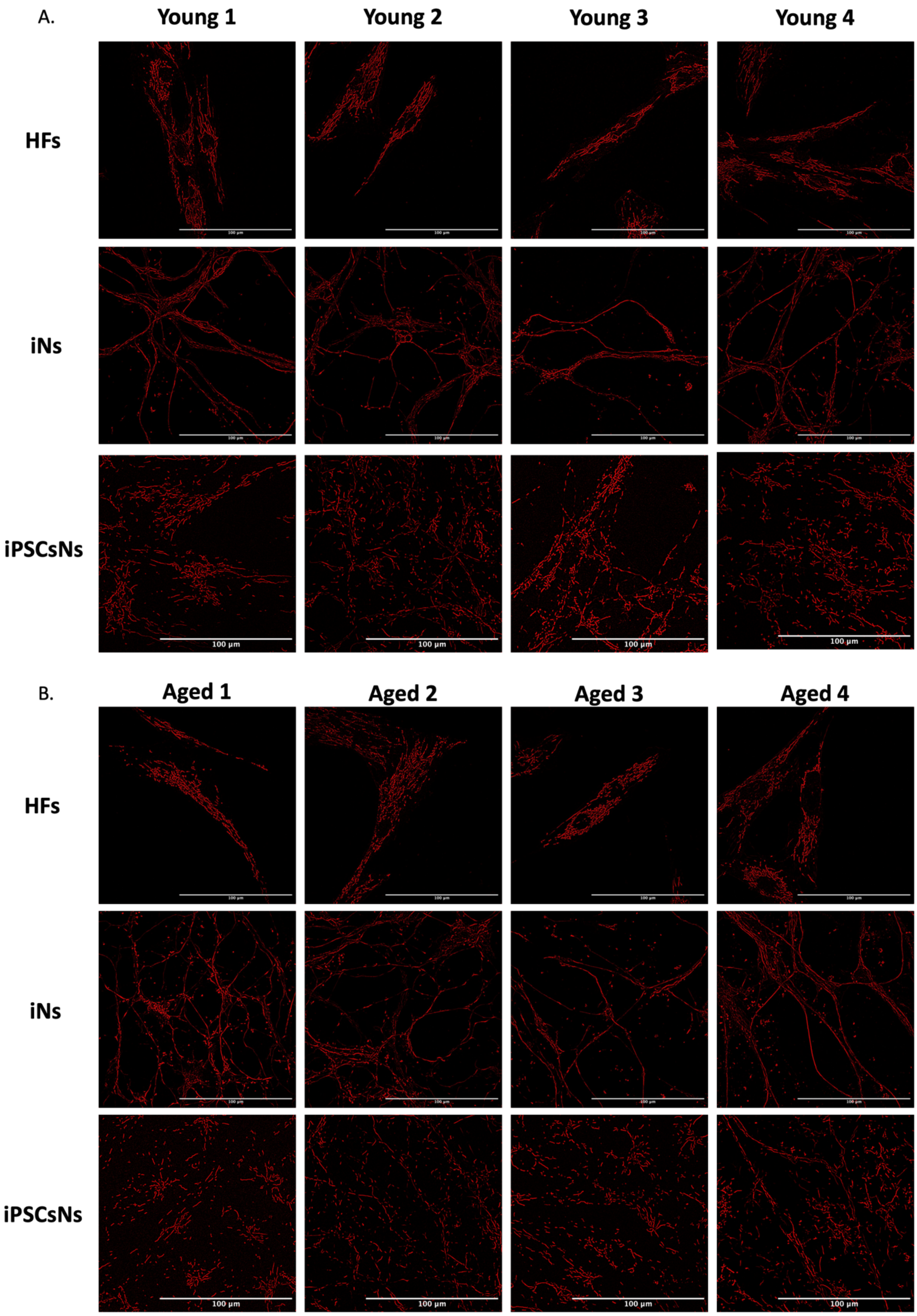

Figure S8: Representative images of the mitochondrial network morphology of each donor, stained for TOMM20, represents **a**, young and **b**, aged donors in HFs, iNs, and iPSCsNs states.
