# Supplementary material for: Tracing mitochondrial marks of neuronal aging in iPSCs-derived neurons and directly converted neurons
Source: Commun Biol. 2025 May 10;8:723. doi: 10.1038/s42003-025-08152-2 (PMC12064796; doi:10.1038/s42003-025-08152-2)
Supplement: Supplementary file 2 — Description of Additional Supplementary Files [file 42003_2025_8152_MOESM2_ESM.docx]

Description of Additional Supplementary Files

File name: Supplementary Data 1
Description: Determination of NAD+ to NADH ratio.

File name: Supplementary Data 2
Description: Statistical parameters: Young versus aged across all experiments

File name: Supplementary Data 3
Description: Statistical parameters of HFs for each donor across all experiments

File name: Supplementary Data 4
Description: Statistical parameters of iNs for each donor across all experiments

File name: Supplementary Data 5
Description: Statistical parameters of iPSCsNs for each donor across all experiments

File name: Supplementary Data 6
Description: Statistical parameters of iPSCs for each donor across all experiments
