# Supplementary material for: Tracing mitochondrial marks of neuronal aging in iPSCs-derived neurons and directly converted neurons
Source: Commun Biol. 2025 May 10;8:723. doi: 10.1038/s42003-025-08152-2 (PMC12064796; doi:10.1038/s42003-025-08152-2)
Supplement: Supplementary file 4 — Reporting Summary [file 42003_2025_8152_MOESM4_ESM.pdf]

nature portfolio

Corresponding author(s): Anne Eckert

Last updated by author(s): Apr 8, 2025

Reporting Summary

Nature Portfolio wishes to improve the reproducibility of the work that we publish. This form provides structure for consistency and transparency in reporting. For further information on Nature Portfolio policies, see our Editorial Policies and the Editorial Policy Checklist.

Statistics

For all statistical analyses, confirm that the following items are present in the figure legend, table legend, main text, or Methods section.

n/a	Confirmed	
☐	☒	The exact sample size (*n*) for each experimental group/condition, given as a discrete number and unit of measurement
☐	☒	A statement on whether measurements were taken from distinct samples or whether the same sample was measured repeatedly
☐	☒	The statistical test(s) used AND whether they are one- or two-sided<br>*Only common tests should be described solely by name; describe more complex techniques in the Methods section.*
☒	☐	A description of all covariates tested
☒	☐	A description of any assumptions or corrections, such as tests of normality and adjustment for multiple comparisons
☐	☒	A full description of the statistical parameters including central tendency (e.g. means) or other basic estimates (e.g. regression coefficient) AND variation (e.g. standard deviation) or associated estimates of uncertainty (e.g. confidence intervals)
☒	☐	For null hypothesis testing, the test statistic (e.g. *F*, *t*, *r*) with confidence intervals, effect sizes, degrees of freedom and *P* value noted<br>*Give P values as exact values whenever suitable.*
☒	☐	For Bayesian analysis, information on the choice of priors and Markov chain Monte Carlo settings
☒	☐	For hierarchical and complex designs, identification of the appropriate level for tests and full reporting of outcomes
☒	☐	Estimates of effect sizes (e.g. Cohen's *d*, Pearson's *r*), indicating how they were calculated

Our web collection on statistics for biologists contains articles on many of the points above.

Software and code

Policy information about availability of computer code

Data collection	The data was collected using the Gen5 software (version 3.11), the Wave Pro Controller software (version 3.0.0.41), and the Step One Plus software (version 2.3).
Data analysis	The data was analysed using Graph Pad Prism 10 (version 10.2.3), Excel software, software R (version 4.2.1), including the packages fmsb (version 0.7.5), and the metaboanalyst.ca website.

For manuscripts utilizing custom algorithms or software that are central to the research but not yet described in published literature, software must be made available to editors and reviewers. We strongly encourage code deposition in a community repository (e.g. GitHub). See the Nature Portfolio guidelines for submitting code & software for further information.

Data

Policy information about availability of data

All manuscripts must include a data availability statement. This statement should provide the following information, where applicable:

- Accession codes, unique identifiers, or web links for publicly available datasets
- A description of any restrictions on data availability
- For clinical datasets or third party data, please ensure that the statement adheres to our policy

The data that support the findings of this study will be openly available after the peer-review process.

Research involving human participants, their data, or biological material

Policy information about studies with human participants or human data. See also policy information about sex, gender (identity/presentation), and sexual orientation and race, ethnicity and racism.

Reporting on sex and gender	Currently, the term "gender" has been used to refer to the biological attribute in the manuscript. We will make the necessary adjustments during the revision process.
Reporting on race, ethnicity, or other socially relevant groupings	We did not incorporate race, ethnicity, or other socially significant factors in our investigation.
Population characteristics	n/a
Recruitment	n/a
Ethics oversight	n/a

Note that full information on the approval of the study protocol must also be provided in the manuscript.

Field-specific reporting

Please select the one below that is the best fit for your research. If you are not sure, read the appropriate sections before making your selection.

☒ Life sciences ☐ Behavioural & social sciences ☐ Ecological, evolutionary & environmental sciences

For a reference copy of the document with all sections, see nature.com/documents/nr-reporting-summary-flat.pdf

Life sciences study design

All studies must disclose on these points even when the disclosure is negative.

Sample size	No sample size calculation was conducted. Instead, the sample size was determined based on the conversion efficiency to iPSCs-derived neurons and directly converted neurons.
Data exclusions	Outliers were identified and excluded through visual inspection.
Replication	All experiments were replicated 3 to 5 times for each condition.
Randomization	Randomization was not applicable to this study due to the small sample size.
Blinding	Blinding was not possible in this study due to the nature of the experimental setup.

Reporting for specific materials, systems and methods

We require information from authors about some types of materials, experimental systems and methods used in many studies. Here, indicate whether each material, system or method listed is relevant to your study. If you are not sure if a list item applies to your research, read the appropriate section before selecting a response.

Materials & experimental systems

n/a	Involved in the study	
☐	☒	Antibodies
☐	☒	Eukaryotic cell lines
☒	☐	Palaeontology and archaeology
☒	☐	Animals and other organisms
☒	☐	Clinical data
☒	☐	Dual use research of concern
☒	☐	Plants

Methods

n/a	Involved in the study	
☒	☐	ChIP-seq
☒	☐	Flow cytometry
☒	☐	MRI-based neuroimaging

Antibodies

Antibodies used	Recombinant Alexa Fluor® 555 Anti-TOMM20 antibody (Abcam, ab221292); Anti-PSA-NCAM-PE, human, mouse, rat (Milteny Biotec, 130-117-394), Anti-MAP2 antibody (Abcam, ab32454), Neuron-specific beta -III Tubulin Antibody (R&D system, MAB1195)

Validation	n/a, recombinant antibodies

Eukaryotic cell lines

Policy information about cell lines and Sex and Gender in Research

Cell line source(s)	The primary human fibroblasts (HFs) and the corresponding iPSCs were purchased from Takara Bio (Kusatsu, Shiga, Japan) or were kindly provided by Dr. Zameel Cader (University of Oxford).
Authentication	The HFs and iPSCs were authenticated either by (Kusatsu, Shiga, Japan) or by Dr. Zameel Cader (University of Oxford) as part of the StemBANCC consortium for biological assays of novel drugs and predictive toxicology. The NPCs and neurons were either FACS sorted or stained for the corresponding markers.
Mycoplasma contamination	All cells were constantly checked for mycoplasma using the MycoAlert® PLUS Mycoplasma Detection Kit and were tested negatively for mycoplasma contamination.
Commonly misidentified lines (See ICLAC register)	Not applicable

Plants

Seed stocks	*Report on the source of all seed stocks or other plant material used. If applicable, state the seed stock centre and catalogue number. If plant specimens were collected from the field, describe the collection location, date and sampling procedures.*
Novel plant genotypes	*Describe the methods by which all novel plant genotypes were produced. This includes those generated by transgenic approaches, gene editing, chemical/radiation-based mutagenesis and hybridization. For transgenic lines, describe the transformation method, the number of independent lines analyzed and the generation upon which experiments were performed. For gene-edited lines, describe the editor used, the endogenous sequence targeted for editing, the targeting guide RNA sequence (if applicable) and how the editor was applied.*
Authentication	*Describe any authentication procedures for each seed stock used or novel genotype generated. Describe any experiments used to assess the effect of a mutation and, where applicable, how potential secondary effects (e.g. second site T-DNA insertions, mosiacism, off-target gene editing) were examined.*
